# Early life dietary intervention in dairy calves results in a long-term reduction in methane emissions

**DOI:** 10.1038/s41598-021-82084-9

**Published:** 2021-02-04

**Authors:** S. J. Meale, M. Popova, C. Saro, C. Martin, A. Bernard, M. Lagree, D. R. Yáñez-Ruiz, H. Boudra, S. Duval, D. P. Morgavi

**Affiliations:** 1 INRAE, UMR Herbivores, VetAgro Sup, Université Clermont Auvergne, 63122 Saint-Genès Champanelle, France; 2 School of Agriculture and Food Sciences, The University of Queensland, Gatton Campus, Gatton, QLD 4343 Australia; 3 Departamento de Producción Animal, Universidad de León, Campus Vegazana s/n, 24071 León, Spain; 4 Plateforme d’Exploration du Métabolisme, MetaboHUB Clermont, Université Clermont Auvergne, 63000 Clermont-Ferrand, France; 5 Estación Experimental del Zaidín (CSIC), Profesor Albareda 1, 18008 Granada, Spain; 6 Research Centre for Animal Nutrition and Health, DSM Nutritional Products, BP 170, 68305 Saint-Louis Cedex, France

**Keywords:** Microbial ecology, Applied microbiology, Microbial communities

## Abstract

Recent evidence suggests that changes in microbial colonization of the rumen prior to weaning may imprint the rumen microbiome and impact phenotypes later in life. We investigated how dietary manipulation from birth influences growth, methane production, and gastrointestinal microbial ecology. At birth, 18 female Holstein and Montbéliarde calves were randomly assigned to either treatment or control (CONT). Treatment was 3-nitrooxypropanol (3-NOP), an investigational anti-methanogenic compound that was administered daily from birth until three weeks post-weaning (week 14). Samples of rumen fluid and faecal content were collected at weeks 1, 4, 11, 14, 23, and 60 of life. Calves were tested for methane emissions using the GreenFeed system during the post-weaning period (week 11–23 and week 56–60 of life). Calf physiological parameters (BW, ADG and individual VFA) were similar across groups throughout the trial. Treated calves showed a persistent reduction in methane emissions (g CH_4_/d) throughout the post-weaning period up to at least 1 year of life, despite treatment ceasing three weeks post-weaning. Similarly, despite variability in the abundance of individual taxa across weeks, the rumen bacterial, archaeal and fungal structure differed between CONT and 3-NOP calves across all weeks, as visualised using sparse-PLS-DA. Similar separation was also observed in the faecal bacterial community. Interestingly, despite modest modifications to the abundance of rumen microbes, the reductive effect of 3-NOP on methane production persisted following cessation of the treatment period, perhaps indicating a differentiation of the ruminal microbial ecosystem or a host response triggered by the treatment in the early development phase.

## Introduction

Due to rising environmental concerns over methane emissions from ruminant production systems, many studies have attempted to target the ruminal microbiota, specifically methanogens, considered responsible. Not only does methane from agriculture account for 14% of total anthropogenic emissions^1^, but it also represents a loss of energy from the animal^2^. However, manipulating the mature ruminal microbiome in favour of reduced greenhouse gas emissions or increased production efficiency has proven challenging. Even following the application of antibiotics or complete rumen content exchanges, the microbiome inevitably shifts back to its pre-intervention state^3^. Recent characterisations of the gastrointestinal microbiome from birth have outlined the sequential establishment of microbial domains^4–6^. This may provide a small window for effective manipulation of methanogens, as ruminal archaea reach levels similar to that of an adult by ~ 14 d of life^7,8^. Abecia et al.^9^ showed the effective manipulation of the microbiome in goats, when the doe was treated in addition to the offspring until three months post weaning. Although the effect did not persist entirely 4 months after, we considered that interventions which target both microbial establishment, and the weaning transition period, may have the potential to imprint the microbiome for reduced methane emissions persisting later into life.

Many studies have examined the use of dietary interventions to modulate the rumen microbiome or an animal’s productive capacity with varying success. For example, the addition of live yeast or certain probiotics can increase feed efficiency via oxygen scavenging^10,11^, whereas, tannins form phenol-dietary protein complexes to improve the flow of digestible bypass protein^12,13^. Strategies to alter the microbiome in favour of reduced methanogenesis include saponins^14,15^, tannins, nitrates and unsaturated fats^16–19^, which exhibit an antiprotozoal effect. However, effects of these interventions have largely failed to persist beyond treatment application periods.

Here we considered the methane inhibitor, 3-nitrooxypropanol (3-NOP), which specifically targets methyl-coenzyme M reductase (MCR), inhibiting catalysis of methane formation during rumen fermentation^20^. In adult ruminants, 3-NOP has effectively inhibited methane by up to 30%, and shown resistance to ruminal adaptation with effects persisting for up to 16 weeks with continuous treatment^21^. However, in a study by Romero-Perez et al.^22^ the effects diminished after treatment ceased, indicating that the inhibiting effects on methane are likely dependent on continuous supplementation of 3-NOP. Importantly, no negative effects have been reported, while a few studies have reported minor improvements in production that could be due to energy being redirected away from methanogenesis^23,24^. As such, we examined 3-NOP as a candidate early-life dietary intervention to modulate microbial establishment in the gastrointestinal tract of dairy calves. Further, we examined the effects of 3-NOP on methane production in post-weaned calves during treatment, and for up to one year post treatment to determine its ability to favour reduced methane production.

## Materials and methods

The experiment was conducted at the INRAE dairy research farm (La boire, Marcenat, https://doi.org/10.15454/1.5572318050509348E12). Procedures were evaluated and approved by the French Ministry of Education and Research (APAFIS #4062-2015043014541577 v5), and carried out in accordance with French and European guidelines and regulations for animal experimentation. Information provided in the manuscript complies with the essential recommendations for reporting of the ARRIVE guidelines.

### Animals, diets and experimental design

Eighteen female Holstein (n = 12), Montbéliarde (n = 4) and Holstein x Montbéliarde (n = 2) calves (42.07 ± 3.85 kg birth weight) were enrolled in the study at birth. Calves were kept with their dam for a few hours but systematically received 2 L of warm colostrum of good quality (≥ 50 g IgG per L) that is conserved at − 20 °C until use. Calves were individually housed for the first week of life and bottle fed 3 L of milk twice daily (0700 h and 1800 h). After the first week, calves were group housed according to treatment with ad libitum access to water and hay. Calves were fed up to 8 L of milk per day through the use of an automated milking system (De Laval, Sursee, Switzerland). Similarly, calves had access to calf starter (STARTIVO, Centraliment, Aurillac, France) from four weeks of age with a maximum daily allowance of 2 kg in the pre-weaning period. In the immediate post-weaning period, calves had access to 2 kg of GENIE ELEVAGE (Centraliment, Aurillac, France). Chemical composition of dietary ingredients is presented in Table S1.

Calves were randomly assigned at birth to either a treatment (3-NOP, 3 mg 3-NOP/kg BW, n = 10 up to week 23, a heifer was removed from the herd due to infertility as a result of being born a twin, i.e., a free-martin, n = 9 at week 60) or control (CONT, placebo premix containing SiO_2_ and propylene glycol only, n = 8, nine calves were recruited but one calf died early in the study) group, such that breed distribution and birthweight were balanced across groups. The 3-NOP supplement contained 10% 3-NOP diluted in propylene glycol and adsorbed on SiO_2_, such that 30 mg of the supplement was fed per kilogram of body weight to achieve the above target dose of 3-NOP. The 3-NOP and placebo were mixed with water (300 mg/mL water) and administered daily via an oral gavage approximately 2 h after feeding. Calves were treated daily from the day of birth, following consumption of colostrum, until 14 weeks of age. All calves were weaned at week 11 using the step-down method over two weeks. After weaning, all calves were group housed in a single pen to replicate normal production practices.

Calves were weighed weekly. Daily individual milk and concentrate intakes prior to weaning were recorded using automated feeders. Total group intake of hay and concentrate, and all refusals in the post-weaning period were recorded twice weekly.

### Sampling

All calves were sampled for rumen fluid and faecal content at 1, 4, 11, 14, 23 and 60 weeks of life. Sampling at week 11 was conducted immediately prior to weaning and sampling at week 14 was conducted just prior to cessation of the treatment. Samples of rumen liquid were obtained via oesophageal tubing at least 2 h after feeding. Aliquots (1 mL) of rumen liquid were immediately frozen in liquid nitrogen and stored at − 80 °C until DNA extraction. Additional rumen liquid aliquots were taken for analysis of volatile fatty acids and ammonia as previously described^25,26^. At week 60, 3 mL of ruminal fluid was added to 3 mL of methyl green formalin saline (MFS) solution (35 mL/L formaldehyde, 0.14 mM NaCl, and 0.92 mM methyl green) and stored in the dark at room temperature until protozoa were counted. At each sampling period, calves were rectally finger-stimulated with sterile-gloved hand to facilitate the collection of a faecal sample, which was immediately frozen in liquid nitrogen and stored at − 80 °C until DNA extraction. Blood samples were taken via jugular venepuncture into a heparin tube at week 11, 14 and 23 for metabolic analysis. Blood was immediately centrifuged at 1500×*g* for 10 min at 4 °C. Plasma was stored at − 80 °C until analysis.

### Methane measurements

Methane emissions were recorded using the GreenFeed system (C-Lock Inc., Rapid City, South Dakota, USA) during two time periods. Firstly, from weaning (week 11) until week 23, one GreenFeed system was programmed using C-Lock Inc. software to deliver a maximum of six rotations of a feed dispensing cup, delivering ~ 6 g of pellet concentrate GENIE ELEVAGE (as fed) per rotation, with intervals of 30 s between each rotation, so that 36 g of pellet was delivered during each visit. During the second phase of measurement when heifers were 57 to 60 weeks of age, two GreenFeed systems were utilised with software programmed to deliver a maximum of six rotations of a feed dispensing cup, delivering approximately 45 g of pellet (as fed) per rotation, with intervals of 30 s between each rotation, so that 270 g of pellet was delivered during each visit. During the second measurement period, calves were separated according to treatment group and allocated to one of two GreenFeed systems. An adaptation period of one week preceded a 4-week experimental recording period. After two weeks, calves were rotated into the alternate pen to eliminate any possible biases between the two GreenFeed systems. During all measurement periods, a minimum of 3 h was required between visits. The calf starter pellets described above were used as an enticement. Recorded methane measurements were included if the total time spent in the feeder was > 3 min with calves visiting the feeder a minimum of three times per day to ensure repeatability of the recorded measurements^27^.

### DNA extraction and amplicon sequencing

Genomic DNA (gDNA) was extracted from each rumen and faeces sample using a bead-beating and on-column purification^28^. DNA extracts were quantified on a Nanodrop 1000 Spectrophotometer (Thermo Fisher Scientific, France) and run on a FlashGel System (Lonza, Rockland, Inc.) to check integrity. Approximately 15 µg of rumen or faecal gDNA were sent to Roy J. Carver Biotechnology Center (Urbana, IL61801, USA) for microfluidics PCR amplification (Biomark HD, Fluidigm, South San Francisco, USA) and HiSeq Illumina paired end sequencing. Selected primers for amplification targeting the V3–V5 region of 16S rRNA gene of bacteria, 16S rRNA gene of archaea, fungal ITS2 and protozoal 18S rRNA gene are presented in Table S2. After amplification all samples were pooled. The library was sequenced on one lane of a HiSeq V2 Rapid flowcell for 251 cycles from each end of the fragments using a HiSeq 500-cycle SBS sequencing kit version 2 (Illumina, San Diego, USA).

### Bioinformatic analysis

All pipelines have a quality control step, removing sequences with Phred scores of < 25 and trimming based on expected amplicon lengths, merging paired reads, chimera identification and removal, and OTU picking. Rare OTUs (tripletons or less) were removed from the total OTU dataset. All sequences were submitted to the Sequence Read Archive under BioProject ID PRJNA641997.

Archaeal output fastq files were analysed by downstream computational pipelines (QIIME software v.1.33.2^29^; length ≥ 250, maximum five primer mismatches, < 8 homopolymers). Chimeric sequences were detected using the UCHIME algorithm (USEARCH 6.1) to run both de novo and reference based chimera detection. Sequences were clustered at the 99% sequence similarity level using GreenGenes v13.8 database using an open reference-based OTU picking approach with the QIIME algorithm and usearch61 method with default parameters^30,31^. For rumen, 2,554,377 reads were obtained, average 20,272 per sample, 87.2% passed quality control and 7.8% chimeric sequences were eliminated. The filtered OTU table had 7987 picked OTUs. For faeces, 1,497,269 reads were obtained, average 13,368 per sample, 97.7% passed quality control and 5.0% chimeric sequences were eliminated. The filtered table had 1380 picked OTUs.

For bacteria and protozoa and for fungi we followed default settings of IM Tornado and PIPITs pipelines, respectively. Raw bacterial (1,929,957 total and 15,317 average per sample; and 2,760,706 reads, average 24,649 per sample, for rumen and faeces, respectively) and protozoal (1,567,407 total and 12,440 average per rumen sample) reads were analysed using the IM-Tornado pipeline^32^, a tool designed to analyse sequencing data producing two separate reads that do not overlap. For rumen contents 90.2% and 87.3% of the reads passed the quality trimming for bacteria and protozoa, respectively; for faecal contents, the good quality reads represented 88.9% and 91.6%. As reads are not overlapping, IM Tornado is performing analysis simultaneously on Read1, Read2 and merged R1R2. At the dereplication step, all quality trimmed reads were collapsed into a set of unique reads which accounted for (average of Read1, Read2 and R1R2) 28 ± 8% and 25 ± 8% in the bacterial rumen and faecal datasets and 30 ± 9% and 16 ± 7% for the protozoa. In all datasets, chimeric sequences represented less than 1%. Silva database release 123_1 was used in both cases to assign taxonomy. OTU picking resulted in 3551 OTUs for rumen bacteria, 2303 for faecal bacteria, 2303 for rumen protozoa and 465 for faecal protozoa. Faecal protozoa OTUs were not associated to known symbiotic ciliates associated to feed degradation. As a preliminary analysis revealed no differences between groups, they are not further presented in the Results section.

Fungi ITS2 fastq files (4,402,028 total reads and an average 34,937 per sample; and 2,211,525 reads, average 19,746 per sample, for rumen and faeces, respectively) were analysed downstream using the automated PIPITS pipeline (https://github.com/hsgweon/pipits^33^). Quality filtered reads represented 96.1% and 95.4% in rumen and faecal contents, respectively. In both datasets 82% of the reads were taxonomically annotated. OTUs were picked using open-reference OTU picking method and the its_12_11_otus reference taxonomy, provided by the UNITE database (https://unite.ut.ee). For rumen 30,100 OTUs were picked and 16,390 OTUs were picked for faeces.

### Absolute quantification of archaeal and bacterial gene copies in ruminal contents

Gene copies of 16S rDNA for archaea and bacteria in week 11, 14, 23 and 60 weeks of life were quantified using a qPCR approach. Primers used are summarized in Table S2; reaction assay and temperature cycles for archaeal and bacterial 16S rDNA were conducted as previously described^26,34^. Triplicate qPCR quantification was performed on 20 ng of extracted gDNA. Amplifications were carried out using SYBR Premix Ex Taq (TaKaRa Bio Inc., Otsu, Japan) on a StepOne system (Applied Biosystems, Courtabeuf, France). Absolute quantification of 16S rDNA copies involved the use of standard curves prepared with gDNA of *Methanobrevibacter ruminantium* DSM 1093 and *Prevotella bryantii* DSM 11,371 as described^34^. Results are expressed as log_10_ gene copies/mL of rumen liquid. PCR efficiencies were 104% and 95% for archaeal and bacterial 16S rDNA, respectively.

### Plasma metabolite analysis

Plasma samples (100 µl) for week 14 and 23 were thawed at room temperature and deproteinised with 300 µl of cold methanol. After centrifugation (14,000×*g*, 10 min, + 4 °C), supernatants were evaporated using a Genevac EZ-2 evaporator (Genevac SP Scientific, Ipswich, UK), and dried residues were dissolved in 50/50 (v/v) acetonitrile/water containing 0.1% formic acid. The mixture was transferred to an autosampler vial, and 10 µl were injected into a LC–MS/ToF system. To evaluate the system’s reproducibility and stability, nine quality-control (QC) samples were prepared by mixing equal volumes of all plasma samples. These QCs were analysed every ten samples, i.e. three times throughout the LC–MS analytical run. Metabolic profiles were then analysed using fast liquid chromatography (1200 Series, Agilent, France) coupled to a time-of-flight mass spectrometer (microTOF, Bruker, Germany). Separation was performed on an Acquity HSS T3 column (Waters, France) using a water/acetonitrile (both containing 0.1% of formic acid) gradient at a flow rate of 0.4 mL/min. The linear gradient of 0% to 100% of acetonitrile was applied for 13 min before returning to the initial conditions and equilibrating for 7 min.

The MS system was operated in positive ionization mode with a scan range of 50–800 m/z. The capillary was set to − 4.5 kV, the nebulizer was operated at 2 bars; the dry gas was set to 8 L/min at a temperature of 200 °C. The capillary exit was set to 90 V with skimmer 1 set to 30 V. The time of flight (ToF) was calibrated by using lithium formate (ions at m/z 90 and 800). For accurate mass acquisition, a formate acetate solution was infused during the run at a flow rate of 100 μL/min monitoring for positive ion mode.

### Dietary chemical composition

Dietary samples of hay and starter concentrate were ground to pass through a 1-mm sieve and stored at 4 °C for analysis according to the Association of Official Analytical Chemists^35^. Organic matter was determined by ashing at 550 °C for 6 h (Association of Official Analytical Chemists [AOAC] method number 923.03). Crude protein was determined by the Dumas method (CP; N × 6.25; AOAC method number 992.15). Cell wall components (NDF and ADF) were determined with residual ash (AOAC methods number 200.04 and 973.18) and starch was determined by a polarimetric method^36^.

### Statistical analysis

Calf growth, intake, physiological parameters (VFA and methane production) were tested using PROC mixed in SAS 9.4 (SAS Institute Inc., Cary, NC, United States) with repeated measures analysis. Means were compared using the LSMEANS/DIFF statement with treatment, week, and the interaction of treatment × week as fixed terms; calves nested within treatment as a random effect and week as a repeated measure. The inclusion of breed in the model was not significant and thus, it was not included. Initial and final BW were analysed using a model similar to that described above, but excluding week as a repeated measure. Alpha diversity metrics: Shannon and Simpson diversity indices, richness, and evenness for all microbial communities and qPCR abundance were analysed independently for each time point using the PROC GLM procedure in SAS.

OTUs tables per week and microbial group were uploaded separately in R, and OTU tables were TSS (Total Sum Scaling) normalized after addition of pseudocounts to the whole datasets to deal with zero-values. Subsequent analysis on these normalized tables was performed using mixOmics^37^ on centred log-ratio (CLR) transformed compositional data. Initial PLS-DA analysis was done on 10 components for performance assessment. Distance and number of components were manually tuned by microbial group and by week, after examining the error rates (perf function). Tuning of the sparse-PLS-DA allowed to select the number of components and variables for the final model. Further, in an attempt to explain fermentation parameters and methane production with respect to OTU abundance, heatmaps were made (cim function on mixOmics) based on the sparse-PLS results on three components. The canonical mode was used with a threshold value of 0.7 and default Euclidian distance and Ward linkage.

Microbial community composition was compared across treatments in each week using a t-test and where appropriate a post hoc FDR test was used to eliminate false significant data^38^. For plasma metabolite analysis, MS data were converted to NetCDF format using DataAnalysis 3.4 software (Bruker Biospin, Germany), and processed using a Galaxy web instance workflow for metabolomics (W4M)^39^. The data were first filtered on retention time, and signals outside the range (< 0.4 min or > 22 min) were removed. Background noise was also removed by subtracting masses found in blank samples (solvents). Signal intensities were then normalized within-batch using a linear regression model^40^. After filtration and normalisation, the number of features was reduced from 2426 to 1175. A data matrix containing mass and retention time with associated signal intensities for all detected peaks was generated and analysed by multivariate analysis using SIMCA-P software (Umetrics, v. 13.01, Sweden). Principal component analysis (PCA) was performed to look for clusters between treatments or identify potential outliers. A tight clustering of QC samples was obtained in a PCA model indicating stable analytical conditions throughout all measurements.

## Results

### Animal growth and rumen physiological parameters remain unchanged

Calf performance data are presented in Table 1. Supplementation with 3-NOP did not affect body weight (BW, kg), average daily gain (ADG), or pre-weaning concentrate or milk intake (*P* ≥ 0.154). Similarly, the inhibitor 3-NOP did not affect individual proportions of rumen volatile fatty acids (VFA; Table S3), or total VFA concentration (mmol/L). Individual proportions of ruminal acetate, propionate, butyrate and the ratio of acetate-to-propionate, in addition to ruminal NH_3_ (mM) were affected by increasing animal age (*P* ≤ 0.013; Table S3).

**Table 1:** Live weight, growth and pre-weaning intake of dairy calves supplemented with 3-NOP (3 mg/kg BW) until 60 weeks of age.

Parameter	CONT	3-NOP	P-value		
			TRT	Week	TRT*Week
**Live weight, kg**					
Birth	40.8 ± 3.96	43.4 ± 3.74	0.154		
Week 23	175.7 ± 4.34	172.1 ± 4.17	0.633		
Week 57	361 ± 12.5	347 ± 11.8	0.434		
Week 60	382 ± 11.9	360 ± 11.2	0.179		
Week 0 to 23	105.7 ± 3.55	104.4 ± 3.35	0.807	0.001	0.997
Week 57 to 60	370.1 ± 9.22	354.2 ± 8.90	0.235	0.320	0.422
ADG week 0 to 23, kg^a^	0.83 ± 0.04	0.79 ± 0.04	0.398	0.001	0.998
ADG week 57 to 60, kg	0.76 ± 0.22	0.56 ± 0.19	0.476	0.034	0.689
**Pre-weaning**					
Concentrate intake, g/d^b^	466 ± 47.3	374 ± 42.3	0.167	< 0.001	0.579
Milk intake, L/d	6.57 ± 0.09	6.45 ± 0.08	0.212	< 0.001	0.700

Values are presented as means ± SE.

n = 8 in CONT; n = 10 in 3-NOP group up to week 23, n = 9 for week 57 to 60.

^a^Average daily gain, ADG.

^b^Intake of hay was not determined on an individual basis as it was offered to the entire group, concentrate intakes may reflect variability in hay intake in young ruminants.

### Short-term treatment in early life reduces methane emission from 1-year old calves

**Figure 1:**
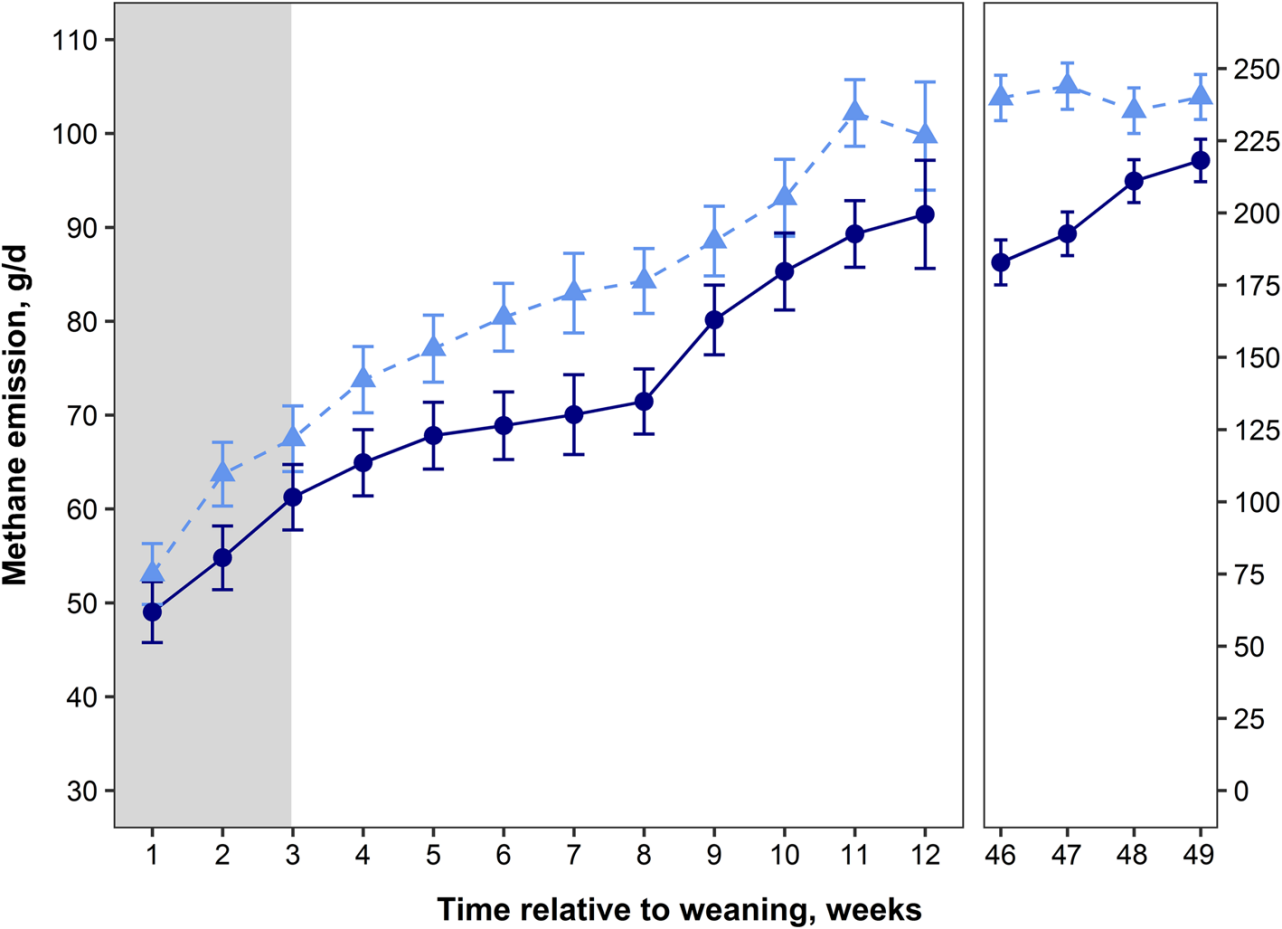
Methane emissions (g CH_4_/d) of weaned calves were persistently reduced with supplementation of 3-NOP. Control calves are represented by the light blue dashed line with triangles. Calves supplemented with 3-NOP until 14 weeks of life are represented by the navy blue line with circles. The grey shaded area represents the post-weaning treatment period. Methane emissions were measured using the GreenFeed System (C-Lock, Inc.). Data are treatment means and bars represent SE (n = 8 in CONT; n = 10 in 3-NOP group up to week 23, n = 9 for the measurement at 46–49 weeks post-weaning (57–60 weeks of life) due to the removal of a 3-NOP heifer from the herd due to infertility as a result of being born a twin, i.e., a free-martin).

Calves receiving 3-NOP showed a persistent reduction in methane emissions (g CH_4_/d) throughout the immediate post-weaning period and at one year post-weaning, despite treatment ceasing at three weeks after weaning (14 weeks of life; Fig. 1). While calves received 3-NOP in the immediate post-weaning period, methane emissions were 10.4% lower (*P* < 0.001) in 3-NOP, compared to CONT calves. A reduction (*P* < 0.001) of 11.9% persisted until week 23. Subsequently, average methane emissions in the immediate post-weaning period were 11.6% lower (*P* < 0.001) in 3-NOP calves, compared to CONT calves (71.2 ± 1.5 vs. 80.5 ± 2.0 g CH_4_/d, respectively). Methane emissions measured around 1 year post-weaning (57 to 60 weeks of age) were 17.5% lower (*P* = 0.002) in heifers treated with 3-NOP as calves, averaging 200.1 ± 4.2 versus 242.7 ± 9.6 g CH_4_/d in 3-NOP and CONT heifers, respectively. Methane yield (g CH_4_/kg BW) tended (*P* = 0.08) to be 9.2% lower in 3-NOP calves during the immediate post-weaning, and was 8.7% lower at 1 year post-weaning, compared to CONT calves (*P* = 0.002; Fig. S1).

### GIT microbial communities differ according to methane-emission phenotypes

#### Rumen bacteria and archaea

Bacterial evenness and richness were similar in calves up to 14 weeks of age (Table S4). However, at week 14, the rumen bacterial community in calves supplemented with 3-NOP was less even (*P* = 0.031) and tended (*P* = 0.085) to have lower richness, compared to CONT calves. Similarly, rumen bacteria in 3-NOP calves had lower richness (*P* = 0.021) at week 60, despite no differences observed in 23-week-old calves. Archaeal diversity indices were higher in week 4 for the CONT. Richness was numerically higher in week 11 and 14 with 3-NOP supplementation, but following cessation of treatment, richness was greater in CONT calves at week 23 (*P* = 0.03) and tended (*P* = 0.08) to be greater in week 60. Conversely, archaeal diversity showed lower (*P* = 0.038) evenness in 3-NOP calves at 14 weeks of age, with a similar tendency (*P* ≤ 0.083; Table S4) observed in week 4 and 60. Ruminal anaerobic fungi were similar in richness and evenness across all weeks, with the exception of week 11, in which 3-NOP calves tended (*P* = 0.68) to show greater richness of anaerobic fungi, compared to CONT calves (Table S4).

**Figure 2:**
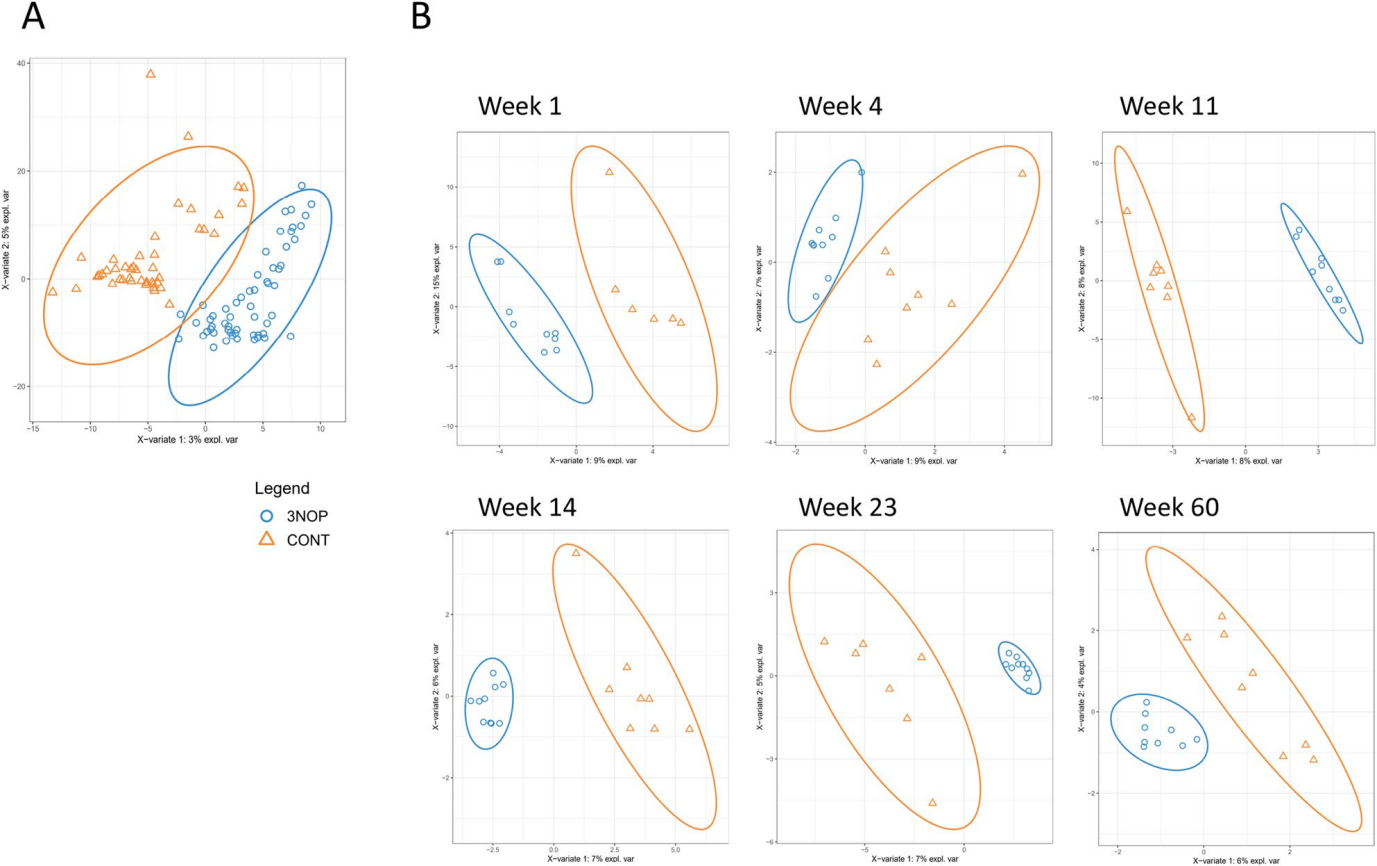
Supplementation of 3-NOP in dairy calves from birth to week 14 of life influences the rumen bacterial community profile. Sparse-PLS-DA loading weights plot of all weeks of ruminal bacteria across treatment groups (CONT, orange vs. 3-NOP, blue) (**A**). Sparse-PLS-DA revealed separation at all sampling weeks between treatment groups (CONT = orange and 3-NOP calves = blue) (**B**).

The rumen microbial community structure, visualized using unsupervised PCA, showed separation among bacteria or archaea by week, reflecting the evolution of the community structure with age, but not by treatment group (Fig. S2 A shows plots for bacteria as an example). To further examine the shifting community structure, we used sparse-PLS-DA to identify discriminant OTUs. Though there was some overlap of treatment groups for the bacterial community, the majority of the CONT samples were separated from the 3-NOP samples (Fig. 2A). The separation was evident when considering the effect of treatment within week, as a clear distinction in bacterial community structure was observed between 3-NOP and CONT calves in every week (Fig. 2B and Fig. S2 B for loading plots and heatmaps). The 3-NOP calves showed less dispersion than CONT calves, particularly in weeks 14, 23 and 60 calves treated with 3-NOP formed a tight cluster.

Separation between CONT and 3-NOP calves revealed several discriminant OTUs and most of them were characteristic of CONT, particularly as calves aged (Fig. 2S B). With some exceptions, discriminant OTUs had low relative abundance and varied between weeks due to age and natural changes in diet. As a consequence, there were no common OTUs defining CONT and 3-NOP groups. Correlation to methane emissions were observed in weeks 23 and 60, but not in week 14 when calves were still treated. In week 23, four OTUs classified as Gastranaerophilales representing 2.1% of sequences were negatively correlated to methane emissions as well as Rikenellaceae_RC9 OTUs that represented 0.7% of bacterial sequences in week 23.

**Figure 3:**
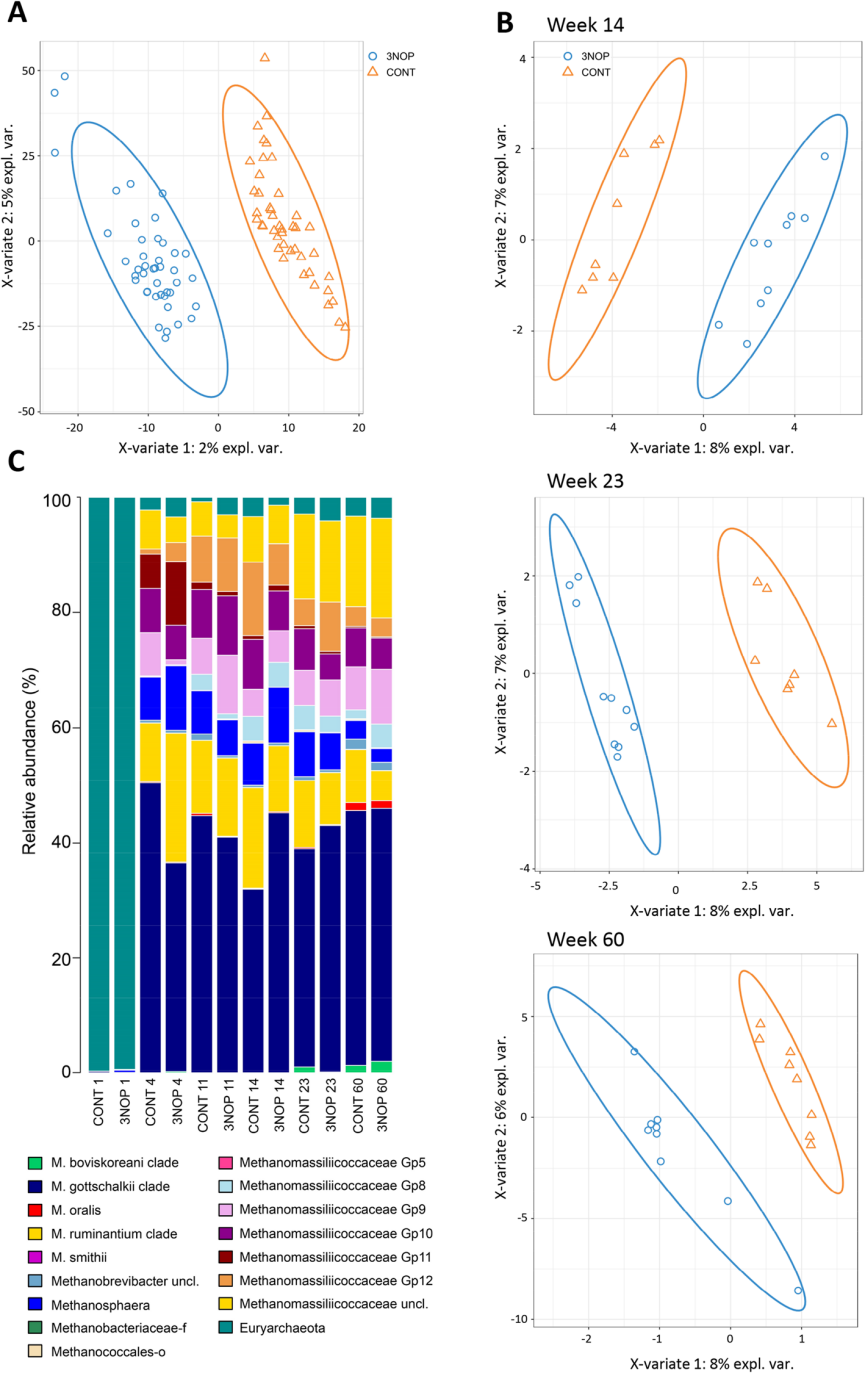
Ruminal archaeal structure differed with 3-NOP supplementation of calves from birth. Sparse-PLS-DA loading weights plot of archaeal structure across all weeks of age showing separation of the two treatment groups, blue = 3-NOP, and orange = CONT(**A**); Sparse-PLS-DA loading weights plots of archaeal microbial structure at week 14, 23 and 60 (**B**), showing clear separation of treatment groups; Relative abundance of archaeal taxa across all weeks (**C**).

Similar sparse-PLS-DA analysis was performed for the rumen archaeal community. However, the fit of the model was unsatisfactory in week 1, and as such, we omitted these results. The rumen archaeal community showed a clear distinction in community structure between 3-NOP and CONT calves when considering calves at all weeks of age, which was further confirmed on a weekly basis (Fig. 3A,B and Fig. S3).

In week 4, OTUs affiliated to *Methanobrevibacter gottschalkii* clade were discriminant in CONT calves, but OTUs were present at a low abundance. The relative abundance of *M. gottschalkii* clade tended (*P* = 0.083) to be lower in 3-NOP calves, at week 4 (Fig. 3C; Table S6). Yet, *M. ruminantium* clade tended to be more abundant (*P* = 0.077) in 3-NOP calves aged 4 weeks. CONT calves were characterized by Methanomassiliicoccaceae Group 9 representing 7.3% of archaeal sequences compared to 0.8% in 3-NOP calves (*P* = 0.002). In weeks 11, 14 and 23 the model identified several discriminant OTUs in CONT and 3-NOP calves, most were associated with the *M. gottschalkii* clade and were linked to both groups of calves. Other discriminant OTUs were associated to *M. ruminantium* clade and Methanomassiliicoccaceae Groups 8, 9, 10 and 12 and these were preferentially linked to CONT calves. In contrast, in week 60 only a single unknown *Methanobrevibacter* OTU was discriminant of CONT calves. As for bacteria, correlations with methane emissions were observed in weeks 23 and 60. It is noted that in week 60 three *Methanomicrobium mobile* representing ~ 3% of archaeal sequences correlated to low methane emissions. Also OTUs associated to Methanomassiliicoccaceae Group 8 were correlated to low methane emissions in week 60 (Fig. S4). *M. mobile* was only detected in week 60 and was more prevalent in 3-NOP calves (*P* < 0.05, Table S6).

Quantification of total ruminal archaea and bacteria by qPCR revealed no statistical differences between treatment groups, in any week (Fig. S5). Similarly, the ratio of archaea-to-bacteria was similar with respect to treatment, across all weeks. However, at 60 weeks of age calves supplemented with 3-NOP had a numerically lower number of Archaea (4.90 vs. 5.08 ± 0.04 log_10_ copies/mL) and ratio of archaea-to-bacteria, compared to CONT calves, respectively (0.75 vs. 0.8 ± 0.01 log_10_ copies/mL; Fig. S5).

#### Rumen fungi and protozoa

Rumen anaerobic fungi showed separation between CONT and 3-NOP calves, particularly in weeks 11, 23 and 60 using sparse-PLS-DA (Fig. S6 A). In week 4, four OTUs affiliated to the Neocallimastigaceae family were discriminant of CONT calves but with low abundances. Conversely, several *Caecomyces communis* OTUs, some with high abundances were associated with 3-NOP calves. In week 11, discriminant OTUs were more diversified in term of taxa and were common across both treatments. After weaning, in week 14, 23, and 60 there were a reduced number of discriminant OTUs (eight or less) that were predominantly associated to with CONT calves (Fig. S6 B). When OTUs are compiled at genera level no differences were observed between groups (Table S6 and Fig. S7). Most OTUs were only assigned at higher taxonomic levels and are not further presented. However, regarding methane, the most abundant discriminant OTUs linked to greater emissions were associated to the genus *Cyllamyces* at 23 weeks and to *Orpinomyces* at week 60.

Ruminal protozoa were only observed at weeks 23 and 60. No differences in alpha-diversity indicators (Table S4) or on counting at 60 weeks (Table S7) were observed between groups. In week 23 the community was dominated by *Entodinium spp.* and was more diversified in older calves at week 60 (Fig. S8 A). The relative abundance of protozoal *Ophryocolex* and unclassified Trichostomatia were greater (*P* ≤ 0.036) in CONT, compared to 3-NOP calves in week 23 (Table S6). In week 60, *Diplodinium* tended (*P* = 0.092) to be more abundant in 3-NOP calves, compared to the CONT. Notwithstanding, sparse-PLS-DA analysis did not show separation between groups (Fig. S8 B).

#### Faecal microbiota

Faecal bacteria are only presented from week 4, due to the low number of samples for 1 week old calves resulting in an unsatisfactory statistical model. Faecal bacteria showed similar (*P* > 0.05) richness and evenness until week 60, where 3-NOP tended (*P* = 0.068) to have greater evenness (Table S5). Sparse-PLS-DA showed bacteria from CONT and 3-NOP calves overlapped in week 4, but from week 11 to week 23 showed clear separation between CONT and 3-NOP calves (Fig. S9 A). Bacteria from week 60 would not converge into a satisfactory sparse-PLS-DA model (not shown), instead we report week 60 bacteria using PLS-DA, which also showed clear separation between treatment groups (Fig. S9 B). In week 14, Rikenellaceae *RC9,* Bacteriodales and *Alloprevotella* were discriminant of the CONT and present at very high abundance. Conversely, 3-NOP was characterised by Porphyromonadaceae (OTU #18) representing 3.28% of sequences. Whereas in week 23, different OTUs belonging to Ruminococcaceae *and* Rikenellaceae *RC9* families were discriminant, but a higher number of sequences for each taxa were present for CONT and 3-NOP, respectively.

The quality of data was not appropriate to perform PLS-DA analysis on faecal archaeal OTUs (data not shown). However, unclassified archaea and unclassified Euryarchaeota comprised the greatest proportion of faecal archaea averaging 38% across all weeks, but varying (*P* ≤ 0.019) between CONT and 3-NOP calves in weeks 4 and 11; and 11 and 14, respectively (Table S8). The relative abundance of *M. gottschalkii* clade was greater (*P* ≤ 0.016) in CONT calves, compared to 3-NOP calves in week 4 and 14, but was similar in all other weeks. Similarly, *M. ruminantium* was greater (*P* ≤ 0.026) in CONT calves, compared to 3-NOP calves in weeks 14 and 60.

Faecal anaerobic fungi in weeks 1 and 4 exhibited a limited number of reads and are therefore not reported. Faecal anaerobic fungi showed no effects of treatment on any alpha-diversity measures (Table S5) or relative abundance (Table S8). Similarly, sparse-PLSDA showed no separation with treatment across week 14 to 60 (data not shown).

### Co-occurrence patterns of ruminal microbes differ by treatment

**Figure 4:**
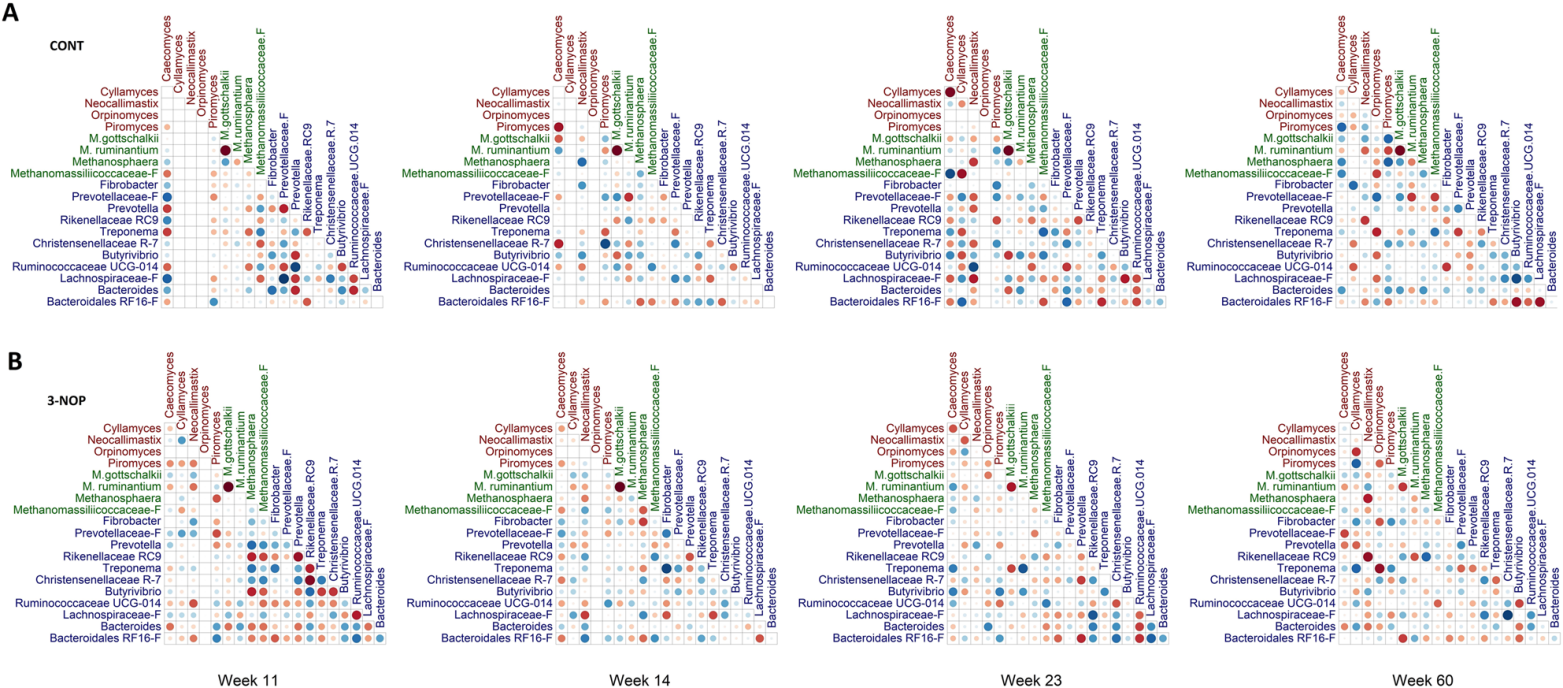
Co-occurrence of ruminal genera differed in CONT versus 3-NOP calves at different ages. Circles represent relationships (positive, blue circles; negative, red circles) between taxa abundance in CONT (**A**) and 3-NOP (**B**) calves, determined using a Spearman non-parametric rank correlation matrix. Larger circles represent stronger correlations. Anaerobic fungi are displayed in red, archaeal taxa in green, and bacteria in blue.

The pattern of microbial co-occurrence in the rumen varied in calves fed CONT versus 3-NOP within each week (Fig. 4). Bacterial taxa *Fibrobacter* showed a strong positive association with *Treponema* (*P* < 0.05) in 3-NOP calves in weeks 11 and 14, but these co-occurrences disappeared after the cessation of 3-NOP treatment and no association between these two taxa were observed in week 23 or 60 of life, or in CONT calves at any age (Fig. 4).

In the archaeal domain, *M. ruminantium* and *M. gottschalkii* clade exhibited a strong negative association in all weeks (*P* ≥ 0.05) regardless of treatment. With these exceptions, no other common associative patterns were observed across weeks.

Comparing between domains revealed differences in the number of significant associations in CONT calves, compared to 3-NOP calves. Calves supplemented with 3-NOP exhibited a mean value of 12.0 ± 0.58 out of a possible 190 associations within and between archaeal, bacterial and fungal taxa each week. Conversely, CONT calves exhibited 8.8 ± 1.11 associations across these domains each week. Interestingly, 3-NOP calves had a greater number of significant associations among bacterial taxa each week (mean 8.3 ± 0.88), compared to in CONT (mean 3.3 ± 0.67) or across domains for week 11, 14 and 23. Yet in week 60, CONT calves had 9 significant associations among bacterial taxa, compared to 3-NOP calves which had 3, resulting in a shift towards an increased number of associations between bacterial and fungal taxa in 3-NOP calves.

### Plasma metabolite profile

The plasma metabolome of CONT and 3-NOP calves was compared at week 14 and 23 by untargeted LC-MS-TOF. In week 14, a PLS-DA model revealed a separation between CONT and 3-NOP calves (Fig. S10 A). The model was acceptable and explained 41% (R^2^X) of variation and had a cross validated predictive ability (Q^2^) of 56%. Conversely, the model in 23 week old calves was poor with no separation between CONT and 3-NOP (Fig. S10 B; Q^2^ = 0.05). Metabolites with variable importance in projection (VIP) values higher than 1 (n = 58) were considered important in discriminating between control and treated calves at 14 weeks. Nine putative candidates were obtained after querying an in-house database containing more than 1000 metabolites analysed under the same conditions. Identification at level 1, confirmed by analytical standards, was obtained for methionine, leucine/isoleucine, tyrosine, and phenylalanine. Whereas, tryptophan, cholic acid glycocholic acid, lyso-phosphatidylcholine (lysoPC16:1) and lyso-phosphatidylethanolamine (lysoPE 18:2) were identified at level 2 (Table S9). The concentration found for these nine metabolites at 14 and 23 weeks is shown in Fig. S11.

## Discussion

The persistence of methane inhibition in calves up to at least one year of age, after 3-NOP treatment ceased at 14 weeks, indicates a robust imprinting effect of the early life intervention. Previous studies have illustrated similar potential. Abecia et al.^9^ showed modifications to the microbiome in goats remained 3 months post-treatment, when the doe was treated in addition to the offspring until two months post-weaning. Similarly, De Barbieri et al.^41^ showed that inoculation of lambs with rumen fluid from sheep in the first 2 weeks of life had effects on rumen bacterial communities lasting up to 5 months of age. However, our study is the first to show successful imprinting for reduced methane emissions. Previous studies on adult ruminants showed reductions in methane emissions using 3-NOP for up to 12 weeks in lactating dairy cows^18^, and 16 weeks in beef cattle^21^. However, once treatment ceased, methane emissions of treated animals or in vitro samples^42^ immediately converged toward the control, eliminating the favourable effects. The differences are not surprising due to the highly resilient nature of the mature ruminal microbiome and the strong host-microbiome interaction, which forces the reversion of the microbial population back to its pre-intervention state^43^. As such, an important contributor to the efficacy of the current intervention was targeting the period of microbial establishment, as well as the weaning transition. These comprise periods of dramatic dietary changes and substantial ruminal maturation and colonization, with large shifts in the rumen and faecal microbiomes^44,45^ and functionality. By targeting both transitional periods, 3-NOP supplementation was able to modulate the rumen environment in dairy calves at a crucial period of development to elicit long lasting effects on methane output. Similarly, the singularity of this result may come from the choice of supplement, due to its very specific microbial target, the active Ni(I) site of the methyl-coenzyme M reductase (MCR) common to all methanogens^20^ that has no redundancy in the ecosystem to compensate its inhibition.

Dietary supplementation of 3-NOP in ruminant livestock has successfully reduced methane emissions in dairy and meat production systems by a mean of 20 to 40%^46^. Importantly, digestibility and production remain largely unaffected by the supplement. Here, methane emissions of dairy calves immediately post-weaning and at one year post-weaning were successfully reduced, whilst the fermentative capacity of the rumen was maintained with 3-NOP supplementation. Evidenced here, and previously, as DMI^47^, ammonia concentration^48^, total VFA^42^, and proportions of propionate and acetate were unaltered^49^. This contrasts previous reports of a reduced A:P ratio with supplementation of 3-NOP^42,47,48^. A possible explanation may be that most studies report larger methane reductions than that observed here. Other explanation could be the overriding effect of calf age and development, where the associated variability in forage:milk:concentrate intake of young calves and establishing microbial structure diminished any treatment effect. Further, hay intake in the young calves was not able to be quantified in the current study, potentially explaining the large variation observed in concentrate intake. Similarly, variability in the degree of methane inhibition observed with 3-NOP is influenced by the method of administration, timing relative to feeding, and the basal diet. The magnitude of methane inhibition (mean 13.1%) in this study from week 11 to 14 during supplementation of weaned calves reinforces that timing of product administration relative to feeding influences the observed effect. The largest reductions have been observed with direct 3-NOP inclusion into the feed ensuring continuous ingestion throughout the meal, in comparison to its application directly into the rumen of cannulated cows^48^.

Yet, as a calf’s digestive tract functions as a pseudo-monogastric in early life, the ideal method of administration, being as a mixture with milk, would induce a reflexive closure of the oesophageal groove, shunting the supplement passed the rumen. We administered the treatment via oral gavage 2 h after milk feeding as a mixture with a minimal amount of water to minimize this instinctive response and to maximise ruminal flow^50,51^. Subsequently, it is possible that the observed effects could be enhanced if an alternative method of administration to young calves is devised. The oesophageal groove reflex is not always stimulated and depending on how milk is provided it can potentially fall in the forestomach^51^. Another approach may be the additional inclusion of 3-NOP with solid feeds; however, this would delay supplementation until calves are ~ 3–4 weeks of age with a highly inconsistent rate of consumption in the initial period, potentially diminishing any effect on the establishment of the ruminal microbiome.

Nevertheless, distinct microbial structures were visible in both the rumen and faeces of CONT and 3-NOP calves, even after cessation of 3-NOP supplementation and group mixing of calves. This indicates a persistent change in the GIT microbiota by the early life intervention. The absence of a consistent modification to the GIT microbiome likely results from a stronger effect of increasing age on microbial community development, than the effect of treatment^45^. Yet, there were some microbial indicators that are known to be associated to enteric methane production. A clear positive association was observed only between *Fibrobacter* and *Treponema* in 3-NOP calves during the treatment period, suggesting a direct impact of 3-NOP on the microbial interactions within the rumen. This altered association between bacterial taxa linked to minimal H_2_ production (*Fibrobacter*), and reduced methane emissions (*Treponema*; as observed by Cunha et al.,^52^), provides support for the effects of 3-NOP to alter the rumen microbial community in favour of a low-methane emitting phenotype. The continued reduction in methane emissions from 3-NOP calves, post-treatment, potentially resulted from an additional suppression of bacterial taxa known to be high H_2_ producers^53^ including numerical reductions on the overall abundance of members of Ruminococcaceae, Christensenellaceae and *Lachnospiraceae* families. This is in agreement with observations by Ramayo-Caldas et al.^54^ in Holstein cattle and Kittelman et al.^55^ in sheep, where higher abundances of Ruminococcaceae, *Coprococcus*, Lachnospiraceae, and Christensenellaceae were linked to higher methane emissions.

Similarly, a negative association between *Fibrobacter* and *M. gottschalkii* clade in week 23 in 3-NOP calves (Fig. 4) supports this, as the production of succinate rather than H_2_ from degradation of cellulose could be expected to reduce the abundance of the hydrogenotrophic *M. gottschalkii* clade. Similar findings have been reported previously, where a lower presence of *M. gottschalkii* clade was linked to lower methane emissions in cattle^38^. Yet, Ramayo-Caldas et al.^54^ noted a lack of clear association between *M. gottschalkii* clade and high or low methane emitting Holstein dairy cows. Importantly, the reliance on bacteria for H_2_ can be inverted, where some species would utilize excess H_2_ available following the supplementation of 3-NOP^56^ reflecting the various pathways through which metabolic hydrogen can be redirected, including formate, succinate, ethanol, microbial biomass and reductive acetogenesis^42^. It was reported that 3-NOP has a greater negative effect on methylotrophic methanogens than on hydrogenotrophic methanogens^57^. This effect was not observed in our study and contrasts with the higher prevalence of Methanomassiliicoccaceae Group 8 that were correlated to lower methane emissions in 3-NOP calves at 60 weeks of age, after treatment ceased.

Such associations between archaea and bacteria also have implications for the reported relationship between the ratio of total archaea to bacteria and methane output and their variability may help explain inconsistencies in the relationship. Due to their key role in methanogenesis, a reduced archaeal abundance would be expected to lower methane emissions. However, this relationship has proven elusive in some studies^55,58–60^, with others reporting only weak correlations^61^. Data on the effects of 3-NOP on rumen microbial structure are scarce and inconsistent. Supplementation of 3-NOP in 6 ruminally-cannulated late-lactation Holstein cows failed to alter methanogenic archaea (*Methanobrevibacter*, *Methanosphaera*, and *Methanomicrobium* spp.) and bacterial cell counts in whole ruminal contents despite a 31% reduction in methane, compared to the control^49^, in agreement with the current results for total quantification of archaea and bacteria by qPCR. However, we noted a numerical reduction in archaeal copy number in 3-NOP calves, compared to CONT calves suggesting that despite a lack of significance potentially resulting from large variation between individual animals, 3-NOP was able to reduce the archaeal population in the rumen at 60 weeks of life. Romero-Pérez et al.^62^ noted no effect of 3-NOP on the total copy number of 16S rRNA genes for methanogens and bacteria in the liquid phase of a RUSITEC, but reported a large decrease in the solid associated methanogens. As the current study was unable to capture solid associated microbes, this may provide an avenue for further investigation. Interestingly, there was an apparent fauna-free status of all calves in the early weeks of life, with protozoa only appearing at week 23 and being present in week 60 of life, likely due to their isolation from adult animals from birth. As protozoa conduct interspecies H_2_ transfer with methanogens, defaunation could potentially reduce methane emissions by 10.5%^63^, yet such an effect would be expected across both CONT and 3-NOP calves, in the current study.

As protozoa, rumen fungi are also involved in interspecies H_2_ transfer with methanogens^64^. They also produce formate, another common substrate for methane synthesis, and might have a role in overall enteric methane emissions^65^. Similarly to bacteria and archaea, the fungal community structure differed between CONT and 3-NOP calves but no divergence in their abundance was detected. Unfortunately, the changes observed in OTUs and correlations to fermentation parameters cannot be associated to the methane phenotype without additional studies.

Metabolomic analysis of plasma revealed a separation between CONT and 3-NOP calves at week 14, while calves were still being treated. However, following cessation of treatment, calves aged 23 weeks showed no separation. This was an exploratory analysis as no additional identification studies were done, but highlights that differences in rumen methane emissions can be reflected in plasma metabolites. It is noted that many of the differences in discriminant metabolites between groups at week 14 showed similar tendencies at week 23 (Fig. S11). Studies with larger number of animals should confirm whether or not differences in metabolic profiles may persist post-treatment.

## Conclusion

Our study examined the neonatal calf up to 60 weeks of life, a period in which the relationship between enteric methane emission and microbial structure has been largely overlooked. We observed a long-term persistent reduction in methane emissions from calves up to 1 year of life following supplementation with 3-NOP from birth, through weaning, to 14 weeks of life. This accounted for an accumulated reduction of circa 150 kg of CO_2_eq that were not released into the environment by calves in their first year of life. In the same way, the ruminal and faecal microbiota of treated and control calves exhibited a clear differentiation, particularly for bacteria. It is apparent that certain microbial taxa were associated with methane emissions. Notwithstanding, contrasting increases or decreases were observed across weeks, indicating that further study is needed to determine the exact role of each taxa, or perhaps that certain taxa are able to modulate their function under certain circumstances. The presence of similar characteristic taxa driving the separation of treatment groups across faeces and ruminal microbiota, and their presence in previous studies, suggests these taxa may be key determinants of methane emissions from dairy calves.

## Supplementary Information

Supplementary Tables.

Supplementary Figures.
