# Supplementary material for: Early life dietary intervention in dairy calves results in a long-term reduction in methane emissions
Source: Sci Rep. 2021 Feb 4;11:3003. doi: 10.1038/s41598-021-82084-9 (PMC7862406; doi:10.1038/s41598-021-82084-9)
Supplement: Supplementary file 1 — Supplementary Tables. [file 41598_2021_82084_MOESM1_ESM.pdf]

Supplementary Material - Tables

Early life dietary intervention in dairy calves results in a long-term reduction in methane emissions

S.J. Meale, M. Popova, C. Saro, C. Martin, A. Bernard, M. Lagree, D.R. Yáñez-Ruiz, H. Boudra, S. Duval, D.P. Morgavi

Table S1. Chemical composition of dietary ingredients fed to all calves (n=18)

	Hay	Starter Concentrate[1]	Concentrate[2]
Dry Matter, %	92.16	91.6	92
N, % DM	1.56	2.78	3.00
NDF, % DM	49.89	21.27	23.2
ADF, % DM	26.08	11.22	13.4
Starch, % DM		23.81	27

[1] Starter concentrate available from 4 weeks up to weaning at 11 weeks

[2] Concentrate supplied after weaning. This pelleted concentrate was also used to lure calves to GreenFeeds.

Table S2. Sequencing and amplicon size of primers used for MiSeq Illumina sequencing and quantative PCR.

Target	Primer name	Sequence (5'-3')	Amplicon size (nt)	Usage	Reference[1]
Archaeal 16S rDNA	Arch349F	GYGCASCAGKCGMGAAW	457	Sequencing	Takai and Horikoshi (2000)
	Arch806R	GGACTACVSGGGTATCTAAT			
Bacterial V3-5 16S rDNA	V3_F357	CCTACGGGAGGCAGCAG	570	Sequencing	Muyzer et al. (1993)
	V5_R926	CCGTCAATTCMTTTRAGT			
Fungal ITS2	ITS3	GCATCGATGAAGAACGCAGC	330	Sequencing	White et al. (1990)
	ITS4	TCCTCCGCTTATTGATATGC			
Protozoal 18S rDNA	F566Euk	CAGCAGCCGCGGTAATTCC	660	Sequencing	Hadziavdic et al. (2007)
	R1200Euk	CCCGTGTTGAGTCAAATTAAGC			
Archaeal 16S rDNA	1406-1389R	AGGAATTGGCGGGGGAGCAC	140	qPCR	Denman et al. (2007)
	896-915F	GAGGAAGGAGTGGACGACGGTA			
Bacterial 16S rDNA	520-F	AGCAGCCGCGGTAAT	280	qPCR	Edwards et al. (2008)
	799-R	CAGGGTATCTAATCCTGTT			

[1]Denman, S.E., Tomkins, N.W., and Mcsweeney, C.S. (2007). *FEMS Microbiology Ecology* 62**,** 313-322.
Edwards, J.E., Huws, S.A., Kim, E.J., and Kingston-Smith, A.H. (2008). *FEMS Microbiology Ecology* 63**,** 141-142.
Hadziavdic, K., Lekang, K., Lanzen, A., Jonassen, I., Thompson, E.M., and Troedsson, C. (2014). *PLOS ONE* 9**,** e87624.
Muyzer, G., De Waal, E.C., and Uitterlinden, A.G. (1993). *Applied and Environmental Microbiology* 59**,** 695-700.
Takai, K., and Horikoshi, K. (2000). *Applied and Environmental Microbiology* 66**,** 5066-5072.
White TJ, Bruns T, Lee S, Taylor JW: Amplification and direct sequencing of fungal ribosomal RNA genes for phylogenetics. PCR Protocols: A Guide to Methods and Applications. Edited by: Innis MA, Gelfand DH, Sninsky JJ, White TJ. 1990, New York: Academic Press Inc, 315-322.

Table S3. Ruminal fermentation parameters in calves supplemented with CONT or 3-NOP up to 60 weeks of age.

	Week 1[1]		Week 4[2]		Week 11[3]		Week 14[2]		Week 23[4]		Week 60[3]			P-values		
Parameter	CONT	3NOP	CONT	3NOP	CONT	3NOP	CONT	3NOP	CONT	3NOP	CONT	3NOP	SE	TRT	Week	TRT*Week
Total VFA, mmol/L	19.57	20.13	60.04	48.65	84.53	87.77	114.01	92.44	70.95	98.43	96.87	120.78	4.275	0.77	<0.001	0.22
Individual VFA, % of total																
Acetate (A)	82.11	78.81	77.35	80.24	72.98	62.97	69.08	70.60	65.90	68.52	73.94	72.41	2.973	0.88	<0.001	0.19
Propionate (P)	14.21	17.30	11.50	11.93	15.61	17.77	19.68	15.14	20.65	18.74	14.38	14.99	0.862	0.43	<0.001	0.29
Butyrate	2.28	2.26	8.59	5.89	9.49	15.82	8.95	11.23	10.53	10.12	10.20	11.05	0.610	0.20	<0.001	0.30
Isovalerate	0.94	1.09	1.33	1.20	0.70	1.56	0.70	1.04	1.07	1.01	0.43	0.43	0.055	0.12	0.013	0.28
Valerate	0.38	0.39	1.05	0.65	0.92	1.42	0.92	1.21	0.72	0.65	0.45	0.49	0.060	0.54	0.011	0.39
Caproate	0.09	0.15	0.19	0.09	0.18	0.37	0.18	0.19	0.26	0.18	0.13	0.13	0.015	0.44	0.003	0.70
A:P ratio	6.16	4.96	6.16	5.91	4.05	3.84	3.60	3.52	4.56	5.20	5.16	4.84	0.196	0.39	0.008	0.27
NH_3, mM	2.54	2.23	14.60	12.15	1.28	1.31	2.28	2.16	4.32	3.06	1.44	1.43	0.470	0.51	0.003	0.44

[1] n=6 in CONT; n=9 in 3-NOP
[2] n=8 in CONT; n=10 in 3-NOP
[3] n=8 in CONT; n=9 in 3-NOP
[4] n=7 in CONT; n=10 in 3-NOP

Table S4. Ruminal alpha diversity metrics in calves supplemented with CONT or 3-NOP up to 60 week of age.

	Week 1[1]				Week 4[2]				Week 11[3]				Week 14[2]				Week 23[4]				Week 60[3]			
	3NOP	CONT	SEM	P-value	3NOP	CONT	SEM	P-value	3NOP	CONT	SEM	P-value	3NOP	CONT	SEM	P-value	3NOP	CONT	SEM	P-value	3NOP	CONT	SEM	P-value
Bacteria																								
Shannon	2.41	2.51	0.126	0.643	3.34	3.70	0.144	0.171	4.46	4.62	0.072	0.102	4.42	4.84	0.102	0.031	4.69	4.76	0.075	0.847	4.03	4.46	0.129	0.124
Simpson	0.75	0.81	0.034	0.728	0.86	0.89	0.025	0.233	0.95	0.96	0.005	0.178	0.95	0.97	0.006	0.019	0.96	0.97	0.004	1.000	0.91	0.93	0.010	0.441
Richness	171	161	9.2	0.418	406	483	24.7	0.102	631	641	16.1	0.386	667	709	15.0	0.085	732	728	14.6	0.700	590	669	18.2	0.021
Evenness	0.47	0.50	0.024	0.817	0.56	0.60	0.023	0.200	0.69	0.72	0.011	0.178	0.68	0.74	0.014	0.031	0.71	0.72	0.011	0.923	0.63	0.68	0.017	0.248
Archaea																								
Shannon	0.40	0.50	0.133	0.754	2.28	2.69	0.082	0.015	2.99	2.93	0.074	0.700	2.95	2.98	0.058	0.825	3.10	3.26	0.046	0.178	3.64	3.60	0.031	0.630
Simpson	0.84	0.70	0.066	0.286	0.80	0.87	0.016	0.058	0.89	0.88	0.012	1.000	0.89	0.91	0.006	0.122	0.90	0.92	0.007	0.211	0.94	0.93	0.003	0.178
Richness	10	18	4.9	0.450	240	312	15.9	0.022	329	311	22.2	0.736	297	251	19.4	0.270	345	400	13.5	0.030	424	476	14.1	0.083
Evenness	0.14	0.23	0.063	0.490	0.42	0.47	0.012	0.070	0.52	0.51	0.009	1.000	0.52	0.54	0.008	0.038	0.53	0.54	0.007	0.564	0.60	0.58	0.004	0.083
Anaerobic fungi																								
Shannon					2.59	2.57	0.084	0.508	4.44	4.17	0.095	0.178	3.97	3.84	0.186	0.508	4.30	4.35	0.105	0.700	4.73	4.89	0.129	0.847
Simpson					0.72	0.73	0.005	0.965	0.95	0.94	0.006	0.211	0.91	0.89	0.021	0.310	0.96	0.96	0.005	0.847	0.97	0.98	0.004	0.248
Richness					524	439	67.2	0.566	723	433	77.1	0.068	560	573	60.5	0.895	691	600	65.1	0.847	735	699	68.6	0.773
Evenness					0.43	0.44	0.011	0.825	0.70	0.70	0.012	0.441	0.64	0.61	0.017	0.354	0.66	0.69	0.010	0.290	0.73	0.76	0.008	0.102
Protozoa																								
Shannon																	0.44	0.77	0.227	0.17	2.53	2.47	0.055	0.32
Simpson																	0.18	0.32	0.102	0.19	0.88	0.87	0.011	0.24
Richness																	15	21	4.5	0.23	41	42	1.2	0.64
Evenness																	0.16	0.24	0.061	0.23	0.68	0.66	0.015	0.24

[1] n=6 in CONT; n=9 in 3-NOP

[2] n=8 in CONT; n=10 in 3-NOP

[3] n=8 in CONT; n=9 in 3-NOP

[4] n=7 in CONT; n=10 in 3-NOP

Table S5. Faecal alpha diversity metrics in calves supplemented with CONT or 3NOP up to 60 week of age.

	Week 1[1]				Week 4[2]				Week 11[2]				Week 14[3]				Week 23[4]				Week 60[2]			
	3NOP	CONT	SEM	P-value	3NOP	CONT	SEM	P-value	3NOP	CONT	SEM	P-value	3NOP	CONT	SEM	P-value	3NOP	CONT	SEM	P-value	3NOP	CONT	SEM	P-value
Bacteria																								
Shannon	2.96	3.01	0.097	1.000	4.21	4.11	0.094	0.56	4.25	4.11	0.067	0.42	4.68	4.71	0.029	0.57	4.53	4.42	0.038	0.18	2.96	3.01	0.097	1
Simpson	0.87	0.86	0.016	0.441	0.95	0.94	0.007	0.70	0.95	0.94	0.005	0.91	0.97	0.97	0.002	0.45	0.96	0.95	0.002	0.054	0.87	0.86	0.016	0.44
Richness	239	257	14.9	0.386	490	494	11.6	0.56	527	525	13.8	1	563	555	10.9	0.76	456	463	6.8	0.50	239	257	14.9	0.39
Evenness	0.54	0.55	0.014	1.000	0.68	0.66	0.014	0.63	0.68	0.66	0.009	0.42	0.74	0.75	0.004	0.45	0.74	0.72	0.006	0.068	0.54	0.55	0.014	1
Anaerobic fungi																								
Shannon					1.04	0.95	0.239	0.69	1.69	1.41	0.353	0.72	3.78	3.69	0.135	0.89	4.08	4.17	0.148	1	1.04	0.95	0.239	0.69
Simpson					0.79	0.82	0.048	0.69	0.86	0.76	0.048	0.35	0.94	0.92	0.009	0.89	0.95	0.94	0.008	0.50	0.79	0.82	0.048	0.69
Richness					30	23	9.8	0.58	156	47	44.2	0.55	422	385	43.3	0.69	358	393	51.5	0.92	30	23	9.8	0.58
Evenness					0.33	0.38	0.084	0.69	0.33	0.38	0.072	0.81	0.63	0.66	0.017	0.31	0.72	0.72	0.010	0.85	0.33	0.38	0.084	0.69

[1] n=6 in CONT; n=9 in 3-NOP
[2] n=8 in CONT; n=9 in 3-NOP
[3] n=6 in CONT; n=9 in 3-NOP
[4] n=8 in CONT; n=10 in 3-NOP

Table S6. Relative abundance of ruminal taxa in calves aged 1, 4, 11, 14, 23 and 60 weeks, as affected by dietary supplementation of 3NOP.

	Week 1[1]		Week 4[2]		Week 11[3]		Week 14[2]		Week 23[4]		Week 60[3]		P-value					
Taxa	CONT	3NOP	CONT	3NOP	CONT	3NOP	CONT	3NOP	CONT	3NOP	CONT	3NOP	1	4	11	14	23	60
**Archaea**																		
Methanobrevibacter	0.68	0.12	62.18	61.41	59.61	56.56	51.12	58.27	52.48	53.04	58.41	53.36	0.262	0.632	0.48	0.057	0.966	0.103
M. boviskoreani clade	0.00	0.00	0.00	0.20	0.00	0.00	0.00	0.00	1.02	0.11	1.28	1.89	NA	0.315	NA	NA	0.301	0.678
M. gottschalkii clade	0.21	0.01	49.31	34.66	43.81	39.77	30.67	44.26	36.60	40.60	42.45	40.90	0.177	0.083	0.59	0.012	0.352	0.686
M. oralis	0.00	0.00	0.20	0.14	0.37	0.15	0.13	0.19	0.22	0.16	1.34	1.22	NA	0.041	0.117	0.182	0.267	0.285
M. ruminantium clade	0.00	0.03	9.97	21.44	12.45	13.18	16.75	11.24	11.18	8.56	8.84	4.88	0.421	0.077	0.847	0.237	0.321	0.024
M. smithii	0.00	0.00	0.01	0.01	0.02	0.01	0.02	0.02	0.03	0.02	0.05	0.03	NA	0.394	0.247	0.994	0.227	0.149
Methanobrevibacter unclassified	0.00	0.00	0.46	0.45	1.07	0.36	0.37	0.44	0.63	0.45	1.69	1.32	NA	0.933	0.026	0.696	0.203	0.131
Methanosphaera	0.00	0.42	7.33	10.68	7.33	6.02	7.03	9.45	7.49	6.03	3.10	2.20	0.435	0.426	0.438	0.297	0.39	0.217
Methanobacteriaceae -F	0.00	0.00	0.01	0.03	0.02	0.01	0.01	0.02	0.02	0.04	0.06	0.04	NA	0.022	0.166	0.399	0.097	0.26
Methanococcales-o	0.05	0.13	0.14	0.11	0.04	0.14	0.36	0.01	0.30	0.06	0.20	0.07	0.414	0.819	0.409	0.234	0.331	0.36
Methanomicrobium mobile	0.00	0.00	0.00	0.00	0.00	0.00	0.00	0.00	0.00	0.00	0.90	3.35	NA	NA	NA	NA	NA	0.036
Methanomassiliicoccaceae																		
Group5	0.00	0.00	0.00	0.00	0.01	0.01	0.01	0.01	0.03	0.02	0.04	0.04	NA	0.132	0.83	0.471	0.133	0.999
Group8	0.00	0.00	0.03	0.02	2.72	0.84	4.07	4.20	4.06	2.63	1.46	3.79	NA	0.647	0.087	0.934	0.444	<0.001
Group9	0.00	0.00	7.30	0.83	6.11	9.84	4.52	5.34	5.87	5.94	7.14	8.83	NA	0.002	0.187	0.626	0.985	0.231
Group10	0.00	0.00	7.47	5.70	8.24	9.95	8.29	6.75	6.93	4.26	6.46	5.04	NA	0.711	0.636	0.749	0.305	0.319
Group11	0.00	0.03	5.88	10.56	1.26	0.72	0.63	0.98	0.50	0.42	0.23	0.17	0.435	0.281	0.498	0.637	0.823	0.55
Group12	0.00	0.00	0.87	3.15	7.86	9.04	12.22	7.07	4.47	8.05	3.28	3.04	NA	0.376	0.652	0.036	0.141	0.73
Methanomassiliicoccaceae unclassified	0.00	0.00	6.60	4.26	5.80	3.89	7.56	6.49	14.21	13.36	15.08	16.09	0.48	0.497	0.244	0.793	0.747	0.631
Euryarchaeota	92.25	99.28	2.17	3.25	0.79	2.97	3.23	1.38	2.82	3.88	3.15	3.40	0.772	0.519	0.244	0.793	0.159	0.437
**Bacteria**																		
Actinobacteria	3.53	5.17	0.88	0.48	0.82	0.82	1.31	0.93	1.71	0.86	1.45	0.63	0.628	0.1	0.989	0.279	0.079	0.043
Actinomyces	2.49	1.43	0.00	0.00	0.00	0.00	0.00	0.00	0.00	0.00	0.00	0.00	0.338	0.749	NA	NA	NA	NA
Atopobium	0.00	0.00	0.17	0.09	0.13	0.09	0.11	0.08	0.12	0.10	0.09	0.04	NA	0.155	0.406	0.421	0.635	0.049
Collinsella	0.52	3.41	0.04	0.06	0.00	0.00	0.00	0.01	0.00	0.00	0.00	0.00	0.405	0.711	NA	0.387	0.42	NA

Coriobacteriaceae-F	0.00	0.05	0.01	0.01	0.04	0.02	0.03	0.03	0.04	0.05	0.05	0.03	0.469	0.896	0.284	0.943	0.253	0.161
Corynebacterium-F	0.08	0.03	0.01	0.00	0.00	0.00	0.14	0.00	0.01	0.00	0.00	0.00	0.253	0.276	0.648	0.273	0.237	NA
Denitrobacterium	0.00	0.00	0.00	0.00	0.00	0.00	0.00	0.01	0.01	0.01	0.00	0.00	NA	0.562	0.989	0.637	0.926	0.595
Eggerthella	0.02	0.02	0.00	0.00	0.00	0.00	0.00	0.00	0.00	0.00	0.00	0.00	0.897	0.648	NA	NA	NA	NA
Enterorhabdus	0.00	0.00	0.02	0.03	0.02	0.03	0.01	0.03	0.01	0.02	0.02	0.01	0.435	0.405	0.165	0.178	0.276	0.306
Olsenella	0.00	0.00	0.57	0.26	0.62	0.63	0.99	0.75	1.49	0.65	1.19	0.49	0.234	0.15	0.969	0.461	0.061	0.07
Paraeggerthella	0.26	0.07	0.00	0.00	0.00	0.00	0.00	0.00	0.00	0.00	0.00	0.00	0.197	NA	NA	NA	NA	NA
Senegalimassilia	0.01	0.02	0.02	0.01	0.02	0.02	0.02	0.01	0.04	0.02	0.07	0.04	0.617	0.51	0.837	0.308	0.272	0.124
Trueperella	0.14	0.12	0.00	0.00	0.00	0.00	0.00	0.00	0.00	0.00	0.00	0.00	0.851	0.276	NA	NA	NA	NA
Bacteroidetes	49.43	61.97	52.01	55.63	37.98	41.87	35.13	35.75	43.62	47.19	39.13	39.39	0.253	0.651	0.338	0.897	0.476	0.954
Alistipes	0.12	0.05	0.00	0.01	0.00	0.00	0.00	0.00	0.00	0.00	0.00	0.00	0.459	0.558	NA	0.387	NA	NA
Alloprevotella	13.26	5.28	0.99	1.47	0.07	0.04	0.08	0.04	0.04	0.04	0.01	0.00	0.07	0.442	0.236	0.024	0.981	0.498
Bacteroidales_BS11_gut_group-F	0.00	0.00	12.99	17.03	0.26	0.16	0.62	0.16	1.42	1.56	1.18	1.26	0.435	0.7	0.065	0.029	0.791	0.919
Bacteroidales_RF16-F	0.00	0.00	4.04	1.21	5.37	8.14	4.64	5.19	13.07	12.02	20.26	20.64	0.28	0.249	0.35	0.786	0.83	0.926
Bacteroidales_S24-7_group	0.06	0.00	0.63	0.81	0.50	2.70	0.58	0.66	0.61	0.41	1.43	0.77	0.237	0.669	0.352	0.761	0.166	0.011
Bacteroidales-O	0.22	0.01	3.94	4.21	3.55	8.70	3.25	2.81	0.16	0.14	0.15	0.20	0.024	0.925	0.019	0.729	0.758	0.206
Bacteroides	21.77	47.19	0.29	0.32	0.00	0.00	0.00	0.01	0.00	0.00	0.00	0.00	0.101	0.898	0.434	0.352	0.632	0.962
Bacteroidetes unclassified	0.00	0.00	0.50	0.09	0.19	0.23	0.14	0.13	0.09	0.25	0.12	0.07	NA	0.291	0.838	0.866	0.339	0.178
Barnesiella	0.02	0.01	0.00	0.02	0.00	0.00	0.00	0.00	0.00	0.00	0.00	0.00	0.405	0.335	NA	NA	NA	NA
Bergeyella	0.01	0.02	0.00	0.00	0.00	0.00	0.00	0.00	0.00	0.00	0.00	0.00	0.534	NA	NA	0.387	0.244	NA
Butyricimonas	3.06	0.79	0.04	0.14	0.00	0.00	0.00	0.00	0.00	0.00	0.00	0.00	0.12	0.319	NA	NA	NA	NA
Capnocytophaga	0.12	0.05	0.00	0.00	0.00	0.00	0.00	0.00	0.00	0.00	0.00	0.00	0.445	NA	NA	0.387	NA	NA
Marinilabiaceae-F	0.00	0.00	0.00	0.00	0.00	0.00	0.00	0.00	0.01	0.00	0.03	0.01	NA	0.276	0.543	0.684	0.147	0.469
Odoribacter	0.43	0.16	0.00	0.01	0.00	0.00	0.00	0.00	0.00	0.00	0.00	0.00	0.027	0.46	NA	NA	NA	NA
Parabacteroides	1.69	0.82	0.00	0.03	0.00	0.00	0.00	0.00	0.00	0.00	0.00	0.00	0.298	0.082	NA	0.387	NA	NA
Phocaeicola	0.00	0.00	1.03	0.39	0.11	0.54	0.11	0.14	0.07	0.07	0.09	0.04	0.234	0.074	0.021	0.582	0.978	0.145
Porphyromonadaceae-F	0.10	z	0.03	0.06	0.00	0.00	0.00	0.00	0.01	0.00	0.02	0.03	0.234	0.396	0.291	0.387	0.091	0.518
Porphyromonas	6.62	1.40	0.01	0.00	0.00	0.00	0.00	0.00	0.00	0.00	0.00	0.00	0.112	0.062	NA	NA	NA	NA
Prevotella	0.87	3.73	20.78	20.89	19.58	12.91	13.25	18.32	18.50	24.85	9.77	10.73	0.294	0.964	0.037	0.9	0.03	0.661
Prevotella_7	0.02	0.34	0.07	0.10	0.10	0.08	4.46	0.18	0.03	0.04	0.00	0.00	0.35	0.7	0.357	0.279	0.488	0.304
Prevotella_9	0.01	0.44	0.00	0.19	0.00	0.00	0.09	0.00	0.00	0.00	0.00	0.00	0.313	0.293	NA	0.136	NA	NA
Prevotellaceae-F	0.90	0.66	2.46	1.94	5.01	3.57	5.04	4.26	3.45	1.93	1.73	1.56	0.623	0.522	0.379	0.702	0.07	0.577

Rikenellaceae_RC9_gut_group	0.05	0.25	4.17	6.64	3.20	4.76	2.84	3.77	6.12	5.86	4.30	4.05	0.335	0.408	0.181	0.303	0.856	0.802
Rikenellaceae-F	0.00	0.00	0.03	0.07	0.02	0.04	0.02	0.06	0.04	0.02	0.03	0.01	0.435	0.347	0.196	0.197	0.553	0.095
Cyanobacteria																		
Gastranaerophilales-O	0.00	0.00	0.47	0.25	5.78	4.45	6.79	3.65	8.25	6.66	4.48	3.91	0.234	0.326	0.466	0.062	0.411	0.531
Elusimicrobia																		
Elusimicrobia-C	0.00	0.00	0.00	0.00	0.01	0.02	0.00	0.00	0.02	0.02	0.07	0.02	NA	0.228	0.621	0.637	0.524	0.251
Fibrobacteres																		
Fibrobacter	0.00	0.25	1.99	6.67	3.73	3.47	7.78	5.90	9.09	6.20	20.75	31.46	0.165	0.126	0.826	0.43	0.04	0.013
Firmicutes	17.10	16.95	28.70	22.04	40.88	38.06	37.24	38.61	20.17	23.10	23.58	15.01	0.987	0.22	0.52	0.745	0.256	0.065
Acetitomaculum	0.01	0.00	5.12	0.65	2.00	1.46	1.83	0.99	0.81	0.61	1.02	0.41	0.449	0.187	0.357	0.103	0.35	0.116
Acidaminococcus	0.00	0.00	0.02	0.01	0.00	0.00	0.00	0.01	0.00	0.00	0.00	0.00	0.435	0.627	NA	0.387	NA	NA
Anaerotruncus	0.01	0.02	0.02	0.01	0.03	0.03	0.02	0.03	0.06	0.04	0.09	0.02	0.423	0.407	0.512	0.326	0.179	4E-04
Anaerovorax	0.00	0.00	0.02	0.02	0.02	0.01	0.02	0.01	0.02	0.01	0.03	0.03	NA	0.928	0.413	0.135	0.024	0.793
Asteroleplasma	0.01	0.01	0.03	0.23	0.09	0.06	0.10	0.16	0.09	0.13	0.14	0.17	0.834	0.408	0.565	0.593	0.682	0.569
Blautia	0.11	0.05	0.02	0.10	0.02	0.01	0.02	0.01	0.02	0.01	0.04	0.02	0.261	0.271	0.577	0.391	0.483	0.141
Butyricicoccus	0.15	0.10	0.00	0.01	0.00	0.00	0.00	0.00	0.00	0.00	0.00	0.00	0.714	0.269	NA	0.387	NA	NA
Butyrivibrio	0.52	0.47	0.92	0.42	2.15	1.74	2.34	1.91	1.29	1.22	1.07	0.78	0.943	0.038	0.563	0.372	0.811	0.37
Catenisphaera	0.00	0.00	0.04	0.05	0.01	0.00	0.01	0.00	0.01	0.00	0.00	0.00	0.234	0.989	0.187	0.552	0.039	0.828
Cellulosilyticum	0.05	0.00	0.00	0.00	0.00	0.00	0.00	0.00	0.00	0.00	0.00	0.00	0.08	NA	NA	NA	NA	NA
Christensenellaceae_R-7_group	0.02	0.01	3.28	1.54	3.03	2.09	2.19	1.99	2.63	2.85	3.40	1.95	0.384	0.122	0.109	0.71	0.736	0.027
Christensenellaceae-F	0.00	0.00	0.02	0.00	0.01	0.01	0.01	0.01	0.01	0.01	0.02	0.01	NA	0.006	0.937	0.558	0.466	0.14
Clostridiales Family_XIII_AD3011_group	0.00	0.00	0.11	0.05	0.03	0.03	0.02	0.03	0.05	0.03	0.05	0.01	NA	0.071	0.982	0.383	0.332	0.047
Clostridiales_Eubacterium_nodatum_group	0.00	0.00	0.03	0.03	0.03	0.02	0.02	0.02	0.02	0.02	0.02	0.01	NA	0.928	0.7	0.766	0.915	0.241
Clostridiales_vadinBB60-F	0.17	0.43	0.46	0.54	0.67	0.49	0.64	1.10	0.75	0.75	1.25	1.37	0.329	0.792	0.327	0.391	0.985	0.667
Clostridiales-O	0.06	0.04	0.03	0.12	0.40	0.08	0.15	0.20	0.03	0.10	0.08	0.04	0.663	0.474	0.288	0.58	0.015	0.004
Clostridium_sensu_stricto_1	0.01	0.02	0.02	1.05	0.00	0.00	0.00	0.00	0.00	0.00	0.00	0.00	0.755	0.051	NA	0.276	0.42	0.148
Clostridium_sensu_stricto_2	0.02	0.01	0.00	0.00	0.00	0.00	0.00	0.00	0.00	0.00	0.00	0.00	0.574	NA	NA	NA	NA	NA
Coprococcus_1	0.00	0.00	0.04	0.05	0.05	0.03	0.02	0.02	0.02	0.04	0.02	0.01	NA	0.879	0.14	0.599	0.105	0.297
Coprococcus_2	0.00	0.00	0.86	0.62	0.56	0.65	0.23	0.34	0.04	0.09	0.01	0.02	NA	0.679	0.789	0.313	0.438	0.495
Defluviitaleaceae_UCG-011	0.00	0.00	0.04	0.01	0.08	0.04	0.05	0.02	0.04	0.03	0.02	0.01	NA	0.008	0.049	0.02	0.415	0.111
Dialister	0.00	0.07	0.00	0.06	0.00	0.00	0.00	0.00	0.00	0.00	0.00	0.00	0.435	0.369	NA	NA	NA	NA

Dielma	0.01	0.03	0.00	0.00	0.00	0.00	0.00	0.00	0.00	0.00	0.00	0.00	0.482	NA	NA	NA	NA	NA
Dorea	0.00	0.02	0.00	0.00	0.00	0.00	0.00	0.00	0.00	0.00	0.00	0.00	0.275	0.877	NA	0.387	NA	NA
Enterococcaceae-F	0.00	0.01	0.00	0.00	0.00	0.00	0.00	0.00	0.00	0.00	0.00	0.00	0.482	NA	NA	NA	NA	NA
Enterococcus	0.01	7.00	0.00	0.00	0.00	0.00	0.00	0.00	0.00	0.00	0.00	0.00	0.428	0.276	NA	NA	NA	NA
Erysipelatoclostridium	0.06	0.05	0.00	0.00	0.00	0.00	0.00	0.00	0.00	0.00	0.00	0.00	0.735	NA	NA	NA	NA	NA
Erysipelotrichaceae_UCG-002	0.00	0.00	1.72	4.28	0.16	0.37	1.10	1.15	0.00	0.00	0.00	0.00	NA	0.502	0.528	0.948	0.42	NA
Erysipelotrichaceae_UCG-004	0.26	0.01	0.30	0.24	0.61	0.52	0.74	1.75	1.41	1.30	3.07	2.57	0.169	0.761	0.579	0.309	0.79	0.195
Erysipelotrichaceae-F	0.00	0.00	0.06	0.03	0.05	0.04	0.03	0.03	0.03	0.03	0.02	0.01	0.488	0.263	0.211	0.955	0.911	0.243
Eubacterium	0.02	0.01	0.01	0.01	0.00	0.00	0.00	0.00	0.00	0.00	0.00	0.00	0.181	0.412	NA	NA	NA	NA
Gemella	0.01	0.01	0.00	0.00	0.00	0.00	0.00	0.00	0.00	0.00	0.00	0.00	0.818	NA	NA	NA	NA	NA
Granulicatella	0.13	0.03	0.00	0.00	0.00	0.00	0.00	0.00	0.00	0.00	0.00	0.00	0.391	0.387	NA	NA	NA	NA
Halanaerobiales-O	0.00	0.00	0.02	0.10	0.04	0.03	0.02	0.03	0.01	0.04	0.00	0.00	NA	0.248	0.231	0.457	0.429	NA
Helcococcus	0.05	0.06	0.00	0.00	0.00	0.00	0.00	0.00	0.00	0.00	0.00	0.00	0.846	0.453	0.362	NA	NA	NA
Howardella	0.00	0.03	0.01	0.01	0.02	0.01	0.02	0.02	0.02	0.03	0.01	0.02	0.105	0.326	0.386	0.479	0.229	0.475
Kandleria	0.00	0.00	0.14	0.18	0.05	0.03	0.03	0.02	0.00	0.00	0.00	0.00	NA	0.834	0.303	0.699	0.259	NA
Lachnobacterium	0.00	0.00	0.01	0.00	0.00	0.00	0.02	0.01	0.00	0.01	0.00	0.00	NA	0.333	0.912	0.258	0.602	0.129
Lachnoclostridium	1.15	0.52	0.05	0.08	0.00	0.00	0.00	0.00	0.00	0.00	0.00	0.00	0.069	0.566	0.188	0.856	0.027	0.176
Lachnoclostridium_10	0.00	0.00	0.04	0.01	0.03	0.02	0.04	0.02	0.05	0.03	0.15	0.07	NA	0.103	0.341	0.263	0.485	0.178
Lachnoclostridium_12	0.00	0.00	0.00	0.00	0.02	0.02	0.03	0.04	0.00	0.01	0.00	0.00	NA	0.276	0.917	0.777	0.182	0.66
Lachnoclostridium_5	0.51	0.16	0.00	0.00	0.00	0.00	0.00	0.00	0.00	0.00	0.00	0.00	0.162	NA	0.304	0.276	0.244	0.592
Lachnospira	0.00	0.00	0.00	0.03	0.02	0.00	0.02	0.03	0.00	0.01	0.00	0.00	NA	0.224	0.299	0.368	0.37	NA
Lachnospiraceae_AC2044_group	0.00	0.01	0.20	0.05	0.82	0.28	0.61	0.89	0.57	0.61	0.84	0.40	0.388	0.198	6E-04	0.349	0.858	0.092
Lachnospiraceae_Bacteroides_pectinophilus_group	0.00	0.00	0.00	0.00	0.00	0.00	0.00	0.00	0.08	0.00	0.00	0.00	NA	NA	NA	NA	0.238	0.304
Lachnospiraceae_Eubacterium_cellulosolvens_group	0.00	0.00	0.06	0.02	0.16	0.05	0.22	0.22	0.02	0.03	0.01	0.01	NA	0.285	0.085	0.954	0.64	0.708
Lachnospiraceae_Eubacterium_eligens_group	0.01	0.01	0.01	0.01	0.07	0.01	0.06	0.05	0.00	0.01	0.00	0.00	0.471	0.962	0.141	0.853	0.322	NA
Lachnospiraceae_Eubacterium_hallii_group	0.00	0.00	0.07	0.32	0.10	0.09	0.14	0.13	0.08	0.07	0.09	0.04	NA	0.313	0.709	0.807	0.733	0.054
Lachnospiraceae_Eubacterium_rectale_group	0.00	0.00	0.02	0.01	0.09	0.03	0.10	0.11	0.02	0.01	0.03	0.02	NA	0.074	0.022	0.907	0.636	0.164

Lachnospiraceae_Eubacterium_ruminantium_group	0.00	0.00	0.12	0.30	0.34	0.53	0.69	0.38	0.10	0.17	0.12	0.08	0.435	0.333	0.462	0.135	0.187	0.314
Lachnospiraceae_Eubacterium_ventriosum_group	0.00	0.00	0.01	0.00	0.03	0.01	0.03	0.04	0.05	0.06	0.10	0.04	NA	0.608	0.015	0.455	0.59	0.029
Lachnospiraceae_Eubacterium_xylanophilum_group	0.00	0.00	0.00	0.01	0.04	0.02	0.06	0.03	0.03	0.04	0.01	0.01	NA	0.389	0.086	0.014	0.608	0.814
Lachnospiraceae_NC2004_group	0.00	0.00	0.00	0.00	0.00	0.03	0.01	0.01	0.00	0.01	0.00	0.00	NA	NA	0.362	0.693	0.14	0.362
Lachnospiraceae_ND3007_group	0.00	0.00	0.00	0.00	0.07	0.01	0.04	0.04	0.05	0.08	0.46	0.34	NA	0.626	0.12	0.98	0.492	0.341
Lachnospiraceae_NK3A20_group	0.00	0.00	3.66	2.09	2.48	1.65	1.39	1.51	0.93	0.82	0.78	0.39	0.071	0.331	0.3	0.849	0.627	0.111
Lachnospiraceae_NK4A136_group	0.01	0.02	0.38	0.44	0.74	3.48	0.48	0.79	0.25	0.26	0.31	0.26	0.469	0.796	0.349	0.247	0.897	0.662
Lachnospiraceae_Ruminococcus_gauvreauii_group	0.00	0.00	0.23	0.28	0.18	0.08	0.22	0.12	0.28	0.19	0.13	0.10	NA	0.778	0.011	0.031	0.118	0.391
Lachnospiraceae_Ruminococcus_torques_group	0.00	0.08	0.00	0.00	0.00	0.00	0.00	0.00	0.00	0.00	0.00	0.00	0.319	0.249	0.177	0.754	NA	NA
Lachnospiraceae_UCG-001	0.00	0.00	0.01	0.01	0.13	0.13	0.04	0.08	0.01	0.02	0.00	0.00	NA	0.99	0.99	0.229	0.185	0.304
Lachnospiraceae_UCG-006	0.00	0.00	0.01	0.01	0.05	0.03	0.05	0.04	0.05	0.05	0.12	0.04	0.435	0.717	0.181	0.368	0.883	0.044
Lachnospiraceae_UCG-008	0.00	0.00	0.04	0.02	0.06	0.06	0.04	0.04	0.04	0.04	0.06	0.03	NA	0.084	0.996	0.852	0.552	0.054
Lachnospiraceae_UCG-009	0.00	0.02	0.03	0.02	0.08	0.03	0.03	0.04	0.05	0.03	0.13	0.05	0.234	0.627	0.163	0.765	0.248	0.109
Lachnospiraceae_XPB1014_group	0.00	0.00	0.76	0.68	0.23	1.16	0.42	0.48	1.41	0.33	0.73	0.28	0.234	0.847	0.108	0.834	0.237	0.103
Lachnospiraceae-F	4.46	0.18	0.90	0.33	1.05	0.72	0.95	0.77	0.62	0.45	1.21	0.74	0.233	0.082	0.111	0.388	0.103	0.258
Lactobacillales-O	0.04	0.02	0.00	0.00	0.00	0.00	0.00	0.00	0.00	0.00	0.00	0.00	0.525	NA	NA	NA	NA	NA
Lactobacillus	0.01	0.00	0.00	0.00	0.00	0.00	0.00	0.02	0.00	0.00	0.00	0.00	0.258	0.74	0.514	0.387	NA	NA
Marvinbryantia	0.00	0.00	0.02	0.01	0.04	0.04	0.03	0.03	0.04	0.05	0.02	0.03	NA	0.215	0.721	0.406	0.645	0.463
Megasphaera	0.00	0.19	0.03	0.02	0.00	0.00	0.00	0.00	0.00	0.00	0.00	0.00	0.435	0.609	NA	NA	NA	NA
Mogibacterium	0.00	0.01	0.06	0.03	0.04	0.02	0.01	0.02	0.07	0.06	0.05	0.06	0.435	0.242	0.153	0.670	0.591	0.761
Moryella	0.00	0.00	0.08	0.14	0.07	0.02	0.03	0.03	0.07	0.03	0.05	0.04	NA	0.348	0.008	0.538	0.009	0.711
Negativicoccus	1.89	2.32	0.00	0.00	0.00	0.00	0.00	0.00	0.00	0.00	0.00	0.00	0.838	NA	NA	NA	NA	NA
Oribacterium	0.00	0.00	0.02	0.04	0.06	0.04	0.54	0.84	0.03	0.07	0.02	0.01	NA	0.492	0.374	0.702	0.396	0.247
Oscillospira	0.00	0.02	0.00	0.00	0.00	0.01	0.00	0.00	0.00	0.01	0.00	0.00	0.421	0.128	0.187	0.202	0.247	NA
Papillibacter	0.00	0.00	0.04	0.05	0.01	0.01	0.02	0.01	0.03	0.02	0.13	0.06	NA	0.892	0.563	0.054	0.904	0.292
Parvimonas	0.85	0.34	0.00	0.00	0.00	0.00	0.00	0.00	0.00	0.00	0.00	0.00	0.484	0.452	NA	NA	0.244	0.304
Peptococcus	0.03	0.01	0.00	0.00	0.00	0.00	0.00	0.00	0.00	0.00	0.00	0.00	0.347	0.276	NA	NA	NA	NA
Peptoniphilus	0.02	0.00	0.00	0.00	0.00	0.00	0.00	0.00	0.00	0.00	0.00	0.00	0.102	NA	NA	NA	NA	NA

Peptostreptococcus	0.11	0.05	0.00	0.00	0.00	0.00	0.00	0.00	0.00	0.00	0.00	0.00	0.147	0.387	NA	0.387	NA	NA
Phascolarctobacterium	0.47	0.43	0.02	0.02	0.00	0.00	0.00	0.00	0.00	0.00	0.00	0.00	0.880	0.873	NA	NA	NA	NA
Pseudobutyrivibrio	0.00	0.03	0.38	0.20	0.84	0.67	0.90	0.62	0.59	0.53	0.72	0.57	0.198	0.015	0.446	0.215	0.645	0.529
Romboutsia	0.01	0.00	0.00	0.00	0.00	0.00	0.01	0.00	0.01	0.00	0.02	0.01	0.078	0.480	0.705	0.326	0.026	0.717
Roseburia	0.01	0.00	0.41	0.17	0.56	0.41	0.65	1.38	0.11	0.14	0.12	0.09	0.234	0.188	0.259	0.441	0.507	0.427
Ruminiclostridium_1	0.00	0.00	0.02	0.00	0.01	0.01	0.02	0.01	0.04	0.02	0.06	0.03	NA	0.107	0.498	0.113	0.228	0.086
Ruminiclostridium_6	0.00	0.00	0.03	0.02	0.02	0.02	0.01	0.01	0.02	0.01	0.00	0.01	0.435	0.694	0.504	0.400	0.444	0.231
Ruminiclostridium_9	0.01	0.01	0.18	0.35	8.68	8.13	7.88	9.20	1.48	5.01	0.03	0.02	0.526	0.272	0.862	0.668	0.052	0.654
Ruminococcaceae_NK4A214_group	0.13	0.11	1.87	1.86	2.39	3.12	2.15	2.67	1.05	1.13	1.04	0.60	0.907	0.990	0.338	0.488	0.797	0.049
Ruminococcaceae_UCG-001	0.00	0.00	0.14	0.02	0.08	0.10	0.10	0.06	0.02	0.02	0.02	0.01	NA	0.161	0.739	0.435	0.894	0.519
Ruminococcaceae_UCG-002	0.01	0.02	0.14	0.34	0.05	0.07	0.06	0.07	0.05	0.03	0.04	0.05	0.688	0.182	0.073	0.704	0.569	0.559
Ruminococcaceae_UCG-004	0.02	0.01	0.01	0.00	0.00	0.00	0.00	0.00	0.00	0.00	0.00	0.00	0.464	0.126	0.391	0.812	0.727	0.988
Ruminococcaceae_UCG-005	0.00	0.02	0.16	0.09	0.12	0.10	0.12	0.07	0.05	0.07	0.14	0.09	0.471	0.168	0.335	0.184	0.481	0.207
Ruminococcaceae_UCG-009	0.00	0.00	0.05	0.03	0.00	0.01	0.00	0.00	0.00	0.00	0.00	0.00	0.234	0.580	0.129	0.104	0.559	NA
Ruminococcaceae_UCG-010	0.00	0.00	0.35	0.14	0.29	0.23	0.19	0.36	0.56	0.40	0.82	0.59	0.136	0.173	0.469	0.281	0.141	0.071
Ruminococcaceae_UCG-011	0.00	0.00	0.01	0.00	0.06	0.02	0.04	0.02	0.08	0.08	0.33	0.04	NA	0.061	0.029	0.078	0.906	0.302
Ruminococcaceae_UCG-012	0.00	0.00	0.01	0.02	0.01	0.01	0.03	0.02	0.08	0.07	0.06	0.03	NA	0.613	0.520	0.387	0.794	0.070
Ruminococcaceae_UCG-013	0.00	0.00	0.02	0.03	0.01	0.01	0.02	0.04	0.04	0.04	0.03	0.02	0.692	0.319	0.554	0.403	0.928	0.194
Ruminococcaceae_UCG-014	0.01	0.00	1.43	1.15	6.06	4.57	5.38	3.74	1.78	1.83	1.19	0.53	0.260	0.658	0.087	0.039	0.948	0.004
Ruminococcaceae_V9D2013_group	0.00	0.00	0.01	0.01	0.01	0.02	0.01	0.02	0.01	0.00	0.01	0.01	NA	0.772	0.838	0.120	0.791	0.668
Ruminococcaceae-F	3.68	1.29	0.27	0.23	0.61	0.58	0.39	0.44	0.20	0.26	0.18	0.14	0.442	0.300	0.830	0.610	0.358	0.379
Ruminococcus_1	0.11	0.76	0.39	0.39	0.92	0.86	0.73	0.76	0.27	0.60	0.34	0.22	0.417	0.968	0.858	0.855	0.017	0.356
Ruminococcus_2	0.00	0.00	0.60	0.55	0.87	0.84	0.50	0.39	0.43	0.77	0.10	0.07	0.206	0.857	0.912	0.483	0.222	0.352
Saccharofermentans	0.00	0.00	1.19	0.18	1.65	1.36	1.37	1.11	0.47	0.75	1.49	1.00	NA	0.036	0.736	0.383	0.149	0.381
Schwartzia	0.00	0.00	0.00	0.01	0.01	0.00	0.01	0.01	0.01	0.01	0.03	0.01	NA	0.762	0.347	0.788	0.515	0.038
Selenomonas	0.00	0.00	0.00	0.14	0.00	0.00	0.00	0.01	0.00	0.00	0.00	0.00	NA	0.098	0.304	0.464	NA	NA
Selenomonas_1	0.00	0.00	0.06	0.08	0.21	0.28	0.19	0.15	0.16	0.13	0.21	0.08	0.435	0.736	0.433	0.464	0.587	0.126
Sharpea	0.00	0.00	0.40	0.22	0.00	0.00	0.11	0.39	0.00	0.00	0.00	0.00	NA	0.578	0.125	0.520	NA	NA
Shuttleworthia	0.00	0.00	0.02	0.01	0.07	0.08	0.10	0.06	0.01	0.01	0.01	0.01	NA	0.735	0.794	0.279	0.504	0.501
Staphylococcus	0.00	0.02	0.01	0.00	0.00	0.00	0.00	0.00	0.00	0.00	0.00	0.00	0.463	0.329	0.574	NA	0.610	NA
Streptococcus	1.63	1.41	0.01	0.01	0.02	0.01	0.03	0.02	0.01	0.01	0.02	0.01	0.898	0.672	0.365	0.866	0.249	0.273
Subdoligranulum	0.02	0.00	0.02	0.02	0.00	0.00	0.00	0.00	0.00	0.00	0.00	0.00	0.095	0.935	NA	NA	NA	NA

Succiniclasticum	0.00	0.00	0.11	0.19	0.09	0.12	0.12	0.09	0.18	0.10	0.42	0.09	NA	0.182	0.508	0.371	0.097	0.072
Syntrophococcus	0.00	0.00	0.03	0.04	0.01	0.01	0.02	0.01	0.01	0.01	0.02	0.01	NA	0.745	0.384	0.105	0.634	0.116
Tyzzerella	0.04	0.21	0.00	0.00	0.00	0.00	0.00	0.00	0.00	0.00	0.00	0.00	0.512	NA	NA	NA	NA	NA
Tyzzerella_3	0.01	0.00	0.02	0.01	0.01	0.02	0.03	0.03	0.02	0.02	0.00	0.00	0.266	0.466	0.499	0.984	0.429	0.412
Tyzzerella_4	0.01	0.05	0.00	0.00	0.00	0.00	0.00	0.00	0.00	0.00	0.00	0.00	0.517	0.276	NA	NA	NA	0.362
Veillonella	0.03	0.06	0.00	0.00	0.00	0.00	0.00	0.00	0.00	0.00	0.00	0.00	0.485	0.105	NA	NA	NA	NA
Veillonellaceae_UCG-001	0.00	0.00	0.01	0.01	0.02	0.01	0.02	0.01	0.03	0.02	0.09	0.03	NA	0.901	0.451	0.655	0.445	0.067
Veillonellaceae-F	0.00	0.00	0.01	0.02	0.04	0.03	0.08	0.05	0.00	0.01	0.01	0.01	NA	0.488	0.695	0.361	0.416	0.437
Fusobacteria	2.44	1.31	0.01	0.01	0.00	0.00	0.02	0.00	0.00	0.00	0.01	0.00	0.368	0.155	0.829	0.281	0.356	0.120
Caviibacter	0.00	0.00	0.01	0.00	0.00	0.00	0.02	0.00	0.00	0.00	0.00	0.00	0.234	0.118	0.862	0.264	0.759	0.993
Fusobacterium	2.43	1.28	0.00	0.00	0.00	0.00	0.00	0.00	0.00	0.00	0.01	0.00	0.364	0.934	0.840	0.714	0.244	0.055
Leptotrichia	0.01	0.03	0.00	0.00	0.00	0.00	0.00	0.00	0.00	0.00	0.00	0.00	0.594	0.450	NA	NA	NA	0.304
Lentisphaerae	0.00	0.00	0.04	0.02	0.02	0.02	0.06	0.03	0.18	0.18	0.62	0.41	0.435	0.393	0.912	0.370	0.995	0.186
Oligosphaeraceae-F	0.00	0.00	0.00	0.00	0.00	0.00	0.00	0.00	0.01	0.00	0.02	0.01	NA	NA	0.869	0.387	0.022	0.113
vadinBE97-F	0.00	0.00	0.03	0.02	0.02	0.02	0.06	0.03	0.17	0.17	0.55	0.36	0.435	0.437	0.980	0.337	0.997	0.155
Victivallaceae-F	0.00	0.00	0.00	0.00	0.00	0.00	0.00	0.00	0.01	0.02	0.04	0.05	NA	0.241	0.435	0.211	0.317	0.739
Proteobacteria	25.77	9.38	11.33	8.73	6.85	6.68	4.55	8.16	9.90	9.69	3.04	2.98	0.021	0.614	0.961	0.189	0.938	0.930
Acinetobacter	0.23	0.03	0.00	0.00	0.00	0.00	0.00	0.00	0.00	0.00	0.00	0.00	0.035	0.500	0.310	0.276	NA	0.362
Actinobacillus	0.25	0.36	0.00	0.00	0.00	0.00	0.00	0.00	0.00	0.00	0.00	0.00	0.719	0.981	NA	0.276	NA	0.647
Alphaproteobacteria-C	0.01	0.00	0.02	0.01	0.04	0.09	0.04	0.07	0.14	0.13	0.03	0.02	0.397	0.233	0.221	0.237	0.886	0.492
Alysiella	0.02	0.01	0.00	0.00	0.00	0.00	0.00	0.00	0.00	0.00	0.00	0.00	0.059	0.244	NA	NA	NA	NA
Betaproteobacteria-C	0.00	0.00	0.03	0.00	0.00	0.00	0.00	0.00	0.00	0.00	0.00	0.00	0.294	0.170	NA	NA	NA	NA
Bradymonadales-O	0.00	0.00	0.00	0.02	0.01	0.00	0.01	0.00	0.00	0.01	0.00	0.01	NA	0.382	0.182	0.169	0.306	0.236
Campylobacter	0.02	0.05	0.01	0.01	0.00	0.00	0.00	0.00	0.00	0.00	0.00	0.00	0.384	0.851	0.153	0.035	NA	NA
Comamonadaceae-F	0.08	0.02	0.00	0.00	0.00	0.00	0.00	0.00	0.00	0.00	0.00	0.00	0.138	0.276	NA	NA	NA	NA
Comamonas	16.08	1.91	0.01	0.00	0.00	0.01	0.00	0.00	0.00	0.00	0.00	0.00	0.037	0.068	0.318	0.276	0.244	NA
Escherichia-Shigella	0.03	0.03	0.00	0.00	0.00	0.00	0.00	0.00	0.00	0.00	0.00	0.00	0.778	0.387	NA	NA	NA	0.304
Gallibacterium	4.85	5.58	0.00	0.00	0.00	0.00	0.00	0.00	0.00	0.00	0.00	0.00	0.749	0.387	NA	0.387	NA	NA
Mannheimia	0.07	0.16	0.00	0.00	0.00	0.00	0.00	0.00	0.00	0.00	0.00	0.00	0.530	0.282	NA	NA	NA	NA
Moraxella	0.04	0.11	0.00	0.00	0.00	0.00	0.00	0.00	0.00	0.00	0.00	0.00	0.606	NA	NA	0.387	NA	NA
Neisseria	0.06	0.07	0.00	0.00	0.00	0.00	0.00	0.00	0.00	0.00	0.00	0.00	0.746	NA	NA	0.276	NA	NA
Neisseriaceae-F	0.07	0.08	0.02	0.04	0.00	0.00	0.00	0.00	0.00	0.00	0.00	0.00	0.940	0.441	NA	0.276	0.420	NA

Parasutterella	0.00	0.01	0.00	0.01	0.00	0.00	0.00	0.00	0.00	0.00	0.00	0.00	0.435	0.176	NA	NA	NA	NA
Pasteurellaceae-F	0.00	0.02	0.00	0.00	0.00	0.00	0.00	0.00	0.00	0.00	0.00	0.00	0.422	0.387	NA	NA	NA	NA
Pseudomonas	0.00	0.00	0.00	0.00	0.01	0.01	0.00	0.00	0.00	0.00	0.00	0.00	NA	0.276	0.663	0.783	NA	0.304
Psychrobacter	0.05	0.07	0.00	0.00	0.00	0.00	0.00	0.00	0.00	0.00	0.00	0.00	0.645	0.202	NA	0.276	NA	NA
Rhodocyclaceae-F	3.90	0.82	2.45	0.41	0.01	0.01	0.02	0.00	0.00	0.00	0.00	0.00	0.365	0.327	0.521	0.011	0.033	0.583
Rhodospirillaceae-F	0.00	0.00	2.95	0.59	6.10	6.33	3.12	7.49	8.86	7.92	2.81	2.70	0.234	0.101	0.948	0.122	0.723	0.873
Rickettsiales-O	0.00	0.00	0.00	0.00	0.02	0.01	0.00	0.08	0.11	0.08	0.11	0.11	NA	0.906	0.896	0.281	0.478	0.985
Ruminobacter	0.00	0.00	0.04	0.05	0.01	0.03	0.01	0.10	0.10	0.06	0.02	0.03	NA	0.790	0.095	0.354	0.493	0.175
Sphingomonas	0.00	0.00	0.00	0.00	0.00	0.00	0.01	0.00	0.00	0.00	0.01	0.02	0.234	0.105	NA	0.368	0.233	0.280
Stenotrophomonas	0.00	0.00	0.00	0.00	0.01	0.01	0.00	0.00	0.00	0.00	0.00	0.00	NA	0.116	0.267	0.387	0.420	0.757
Succinimonas	0.00	0.00	0.00	0.00	0.01	0.01	0.64	0.01	0.01	0.01	0.00	0.02	NA	0.276	0.724	0.281	0.972	0.042
Succinivibrio	0.00	0.01	5.62	6.13	0.00	0.01	0.01	0.02	0.01	0.14	0.02	0.01	0.349	0.907	0.084	0.568	0.378	0.704
Succinivibrionaceae_UCG-001	0.00	0.00	0.04	0.06	0.56	0.03	0.59	0.23	0.00	0.41	0.00	0.00	NA	0.804	0.096	0.251	0.395	NA
Succinivibrionaceae_UCG-002	0.00	0.00	0.02	0.64	0.07	0.06	0.07	0.10	0.64	0.91	0.01	0.02	NA	0.392	0.747	0.580	0.749	0.644
Succinivibrionaceae-F	0.00	0.00	0.07	0.76	0.00	0.00	0.00	0.04	0.00	0.00	0.00	0.00	0.435	0.284	NA	0.407	NA	NA
Sutterella	0.00	0.05	0.00	0.01	0.00	0.03	0.00	0.01	0.01	0.00	0.00	0.01	0.260	0.481	0.091	0.927	0.330	0.602
Saccharibacteria	0.00	0.00	0.00	0.00	0.05	0.04	0.09	0.03	0.06	0.05	0.07	0.03	NA	0.784	0.556	0.025	0.359	0.060
Spirochaetae	1.41	4.90	3.75	5.64	1.92	2.60	4.86	5.19	4.66	4.52	4.52	4.24	0.510	0.521	0.067	0.814	0.910	0.841
Sphaerochaeta	0.02	0.80	0.28	2.30	0.09	0.14	0.08	0.17	0.19	0.14	0.38	0.25	0.436	0.125	0.458	0.328	0.264	0.012
Spirochaeta_2	0.00	0.00	0.01	0.00	0.01	0.00	0.00	0.00	0.03	0.02	0.03	0.02	NA	0.223	0.125	0.698	0.521	0.258
Spirochaetaceae-F	0.00	0.00	0.02	0.00	0.00	0.01	0.00	0.00	0.05	0.02	0.01	0.01	NA	0.276	0.212	0.214	0.294	0.482
Treponema	1.39	4.10	3.44	3.34	1.82	2.46	4.78	5.02	4.40	4.34	4.10	3.96	0.608	0.970	0.101	0.860	0.963	0.924
Tenericutes	0.18	0.02	0.52	0.40	1.55	1.35	1.52	1.22	1.70	1.14	1.83	1.57	0.071	0.423	0.465	0.314	0.080	0.351
Anaeroplasma	0.06	0.00	0.09	0.09	0.34	0.16	0.39	0.30	0.84	0.47	0.65	0.88	0.237	0.933	0.045	0.251	0.018	0.173
Mollicutes-C	0.12	0.01	0.43	0.31	1.20	1.19	1.13	0.93	0.87	0.67	1.19	0.69	0.133	0.338	0.935	0.396	0.329	0.010
Verrucomicrobia	0.00	0.00	0.02	0.00	0.00	0.00	0.00	0.00	0.08	0.02	0.02	0.02	0.173	0.300	0.697	0.950	0.159	0.904
**Anaerobic Fungi**																		
Anaeromyces	0.00	15.98	0.00	0.16	0.00	0.00	0.00	0.00	0.00	0.00	23.59	4.73	0.356	0.335	NA	NA	NA	0.670
Caecomyces	34.67	24.97	59.78	77.99	67.88	64.29	86.95	70.85	27.34	25.82	14.62	12.82	0.738	0.191	0.783	0.209	0.856	0.614
Cyllamyces	0.00	0.00	0.15	1.64	0.00	4.17	0.00	5.62	29.70	30.06	8.48	17.14	NA	0.165	0.362	0.387	0.977	0.513
Neocallimastigaceae-F	17.19	58.22	36.04	15.15	21.37	13.27	6.12	18.97	38.56	36.57	19.79	17.95	0.194	0.083	0.404	0.293	0.778	0.262

Neocallimastix	23.61	0.00	0.07	0.40	0.00	0.01	0.11	0.01	0.74	0.83	0.86	0.70	0.356	0.370	0.185	0.289	0.867	0.613
Orpinomyces	0.00	0.00	0.00	0.01	0.00	0.00	0.00	0.00	0.00	0.51	26.20	40.74	NA	0.396	NA	NA	0.420	0.876
Piromyces	24.53	0.83	3.95	4.66	10.75	18.26	6.82	4.54	3.66	6.21	6.47	5.92	0.370	0.840	0.526	0.494	0.527	0.178
**Protozoa**																		
Dasytricha	-	-	-	-	-	-	-	-	5.82	0.14	5.79	5.36	-	-	-	-	0.172	0.883
Diplodinium	-	-	-	-	-	-	-	-	0.88	4.32	9.19	14.52	-	-	-	-	0.187	0.092
Entodinium	-	-	-	-	-	-	-	-	88.70	94.34	33.05	40.10	-	-	-	-	0.257	0.308
Eremoplastron	-	-	-	-	-	-	-	-	0.16	0.70	1.87	1.64	-	-	-	-	0.498	0.806
Eudiplodinium	-	-	-	-	-	-	-	-	0.02	0.02	6.26	6.69	-	-	-	-	0.405	0.893
Isotricha	-	-	-	-	-	-	-	-	0.00	0.00	5.14	3.83	-	-	-	-	0.861	0.555
Ophryoscolex	-	-	-	-	-	-	-	-	3.61	0.18	19.56	12.77	-	-	-	-	0.036	0.222
Polyplastron	-	-	-	-	-	-	-	-	0.35	0.15	3.08	3.34	-	-	-	-	0.098	0.825
Trichostomatia -C	-	-	-	-	-	-	-	-	0.46	0.15	16.06	11.75	-	-	-	-	0.023	0.100

Taxa are presented at the highest taxonomic level at which they were classified, F- family, O- order, C-class; P-values represent pairwise comparisons between CONT and 3NOP, within each week. Only taxa that were present at >0.01 in at least one group in a least one week are shown.

[1] n=6 in CONT; n=9 in 3-NOP

[2] n=8 in CONT; n=10 in 3-NOP

[3] n=8 in CONT; n=9 in 3-NOP

[4] n=7 in CONT; n=10 in 3-NOP

Table S7. Protozoa concentration in the rumen fluid of calves at 60 weeks of age as affected by dietary supplementation of 3-NOP.

	CONT	3NOP	SEM	P-value
Total protozoa, log_{10} cells/mL	4.96	4.96	0.076	0.98
Entodiniomorphs, log_{10} cells/mL				
Small (<100 μm)	4.92	4.93	0.077	0.92
Large (>100 μm)	3.64	3.48	0.173	0.36
Holotrichs, log_{10} cells/mL				
Dasytricha	3.11	3.20	0.165	0.58
Isotricha	2.54	2.51	0.270	0.93

N= 8 in CONT; n= 9 in 3-NOP

Table S8. Relative abundance of faecal taxa in calves aged 1, 4, 11, 14, 23, and 60 weeks, as affected by dietary supplementation of 3-NOP.

	Week 1[1]		Week 4[2]		Week 11[2]		Week 14[3]		Week 23[4]		Week 60[2]		P-value					
Taxa	CONT	3NOP	CONT	3NOP	CONT	3NOP	CONT	3NOP	CONT	3NOP	CONT	3NOP	1	4	11	14	23	60
**Archaea**																		
Archaea unclassified	63.91	81.77	41.85	78.45	42.30	14.83	26.27	13.77	16.61	18.49	13.91	15.60	0.54	0.016	0.009	0.061	0.70	0.23
Euryarchaeota unclassified	20.94	17.99	21.99	14.90	26.12	62.96	33.25	73.13	41.10	52.77	45.00	49.27	0.92	0.52	0.019	0.001	0.068	0.21
Methanobrevibacter_boviskoreani_clade	0.00	0.00	0.00	0.00	0.00	0.00	0.00	0.00	0.00	0.00	0.00	0.04	NA	NA	NA	NA	NA	0.36
Methanobrevibacter_gottschalkii_clade	15.07	0.01	18.12	0.68	16.31	10.75	12.96	3.83	19.46	11.89	16.52	13.65	0.22	0.016	0.47	0.014	0.13	0.38
Methanobrevibacter_oralis	0.02	0.00	0.00	0.00	0.02	0.00	0.00	0.00	0.01	0.01	0.05	0.03	0.22	0.33	0.28	0.41	0.97	0.43
Methanobrevibacter_ruminantium_clade	0.04	0.00	5.09	0.24	3.73	8.86	15.64	3.68	6.31	5.61	10.11	5.71	0.22	0.17	0.28	0.006	0.64	0.021
Methanobrevibacter_smithii	0.00	0.00	0.00	0.00	0.00	0.00	0.00	0.00	0.00	0.00	0.00	0.00	NA	NA	NA	0.41	0.97	NA
Methanobrevibacter_unclassified	0.02	0.02	0.02	0.01	0.04	0.03	0.04	0.32	0.62	0.38	0.65	0.98	0.81	0.43	0.70	0.20	0.49	0.42
Methanosphaera	0.00	0.00	2.57	1.52	2.08	0.81	7.34	1.56	1.29	0.68	0.58	0.26	NA	0.72	0.25	0.094	0.33	0.17
Methanococcales-o	0.00	0.02	7.24	0.00	8.54	1.42	4.48	3.03	4.99	3.43	4.59	3.97	0.48	0.33	0.18	0.57	0.40	0.42
Methanomassiliicoccaceae_Group10	0.00	0.00	0.00	0.00	0.00	0.00	0.00	0.00	0.00	0.00	0.00	0.00	NA	NA	NA	NA	0.39	0.30
Methanomassiliicoccaceae_Group11	0.00	0.00	0.00	0.00	0.00	0.00	0.00	0.00	0.00	0.00	0.00	0.00	NA	NA	NA	NA	0.53	NA
Methanomassiliicoccaceae_Group12	0.00	0.00	0.01	0.03	0.01	0.00	0.00	0.00	0.00	0.00	0.00	0.00	0.482	0.37	0.37	0.41	NA	0.11
Methanomassiliicoccaceae_Group3	0.00	0.00	0.00	0.00	0.00	0.00	0.00	0.00	0.03	0.01	0.04	0.05	NA	NA	NA	NA	0.046	0.71
Methanomassiliicoccaceae_Group8	0.00	0.18	0.00	0.00	0.02	0.32	0.00	0.38	5.04	3.55	2.73	3.14	0.48	NA	0.38	0.28	0.44	0.68
Methanomassiliicoccaceae_Group9	0.00	0.00	0.00	0.00	0.01	0.01	0.00	0.00	3.32	2.09	4.39	5.15	NA	NA	0.98	NA	0.33	0.51
Methanomassiliicoccaceae_unclasified	0.00	0.00	3.11	4.17	0.83	0.00	0.02	0.01	0.60	0.42	0.82	0.93	NA	0.77	0.30	0.56	0.50	0.54
**Bacteria**																		
Bacteria_unclassified	0.16	0.07	0.01	0.05	0.01	0.01	0.01	0.01	0.06	0.05	0.10	0.08	0.59	0.044	0.89	0.33	0.21	0.50
Actinobacteria	12.07	7.22	0.21	0.08	0.07	0.03	0.05	0.05	0.09	0.10	0.10	0.07	0.60	0.30	0.39	0.98	0.70	0.85
Bacteroidetes	37.54	28.97	58.99	41.58	44.66	43.75	49.06	46.28	44.28	40.14	41.80	39.91	0.52	0.059	0.79	0.48	0.037	0.48
Cyanobacteria	0.00	0.00	1.11	0.15	0.79	1.08	0.08	0.08	0.57	0.76	0.32	0.41	NA	0.22	0.56	0.96	0.44	0.22
Elusimicrobia	0.00	0.00	0.00	0.00	0.06	0.04	0.01	0.01	0.01	0.01	0.02	0.04	NA	0.13	0.59	0.74	0.64	0.16

Fibrobacteres	0.00	0.00	0.00	0.00	0.40	0.00	0.00	0.00	0.01	0.02	0.00	0.00	NA	NA	0.31	0.83	0.50	NA
Firmicutes	38.65	29.36	34.55	30.86	50.78	52.63	44.34	49.64	52.73	56.44	55.09	57.38	0.425	0.52	0.51	0.097	0.067	0.49
Fusobacteria	0.22	9.62	1.30	19.06	0.00	0.00	0.00	0.00	0.00	0.00	0.00	0.00	0.15	0.027	0.36	NA	NA	NA
Lentisphaerae	0.00	0.00	0.00	0.00	0.00	0.01	0.00	0.00	0.02	0.04	0.08	0.11	NA	NA	0.37	0.58	0.15	0.46
Proteobacteria	11.22	24.74	2.91	8.07	0.87	0.89	0.51	0.86	0.68	0.79	0.37	0.44	0.16	0.006	0.96	0.46	0.66	0.75
Saccharibacteria	0.00	0.00	0.00	0.00	0.01	0.00	0.01	0.01	0.01	0.02	0.02	0.01	NA	0.30	0.61	0.84	0.17	0.052
Spirochaetae	0.00	0.00	0.00	0.00	1.55	0.86	5.47	2.33	0.87	0.83	1.66	1.07	NA	NA	0.33	0.12	0.82	0.68
Tenericutes	0.15	0.00	0.92	0.15	0.80	0.56	0.45	0.69	0.65	0.78	0.40	0.41	0.48	0.070	0.49	0.16	0.28	0.67
Verrucomicrobia	0.00	0.00	0.00	0.00	0.00	0.15	0.00	0.02	0.01	0.03	0.05	0.07	NA	NA	0.097	0.16	0.13	0.55
**Anaerobic Fungi**																		
Anaeromyces	-	-	-	-	0.00	0.00	0.00	0.00	0.00	0.00	0.00	3.34	-	-	NA	NA	NA	0.19
Caecomyces	-	-	-	-	19.77	22.19	49.75	75.64	64.01	43.09	33.35	39.86	-	-	0.93	0.41	0.12	0.58
Cyllamyces	-	-	-	-	0.00	0.00	0.00	0.00	9.40	28.36	18.24	9.82	-	-	NA	NA	0.13	0.34
Neocallimastix	-	-	-	-	0.00	0.00	0.00	0.00	0.04	0.00	0.01	0.03	-	-	NA	NA	0.30	0.25
Orpinomyces	-	-	-	-	0.00	0.00	0.00	0.01	0.00	0.00	1.25	0.76	-	-	MA	0.45	NA	0.65
Piromyces	-	-	-	-	0.00	0.31	0.00	0.36	0.53	0.51	1.54	3.29	-	-	0.39	0.24	0.97	0.31
Neocallimastigaceae-F	-	-	-	-	80.23	77.49	50.25	24.00	26.03	28.04	4.19	6.25	-	-	0.91	0.41	0.86	0.36

[1] n=6 in CONT; n=9 in 3-NOP
[2] n=8 in CONT; n=9 in 3-NOP
[3] n=6 in CONT; n=9 in 3-NOP
[4] n=8 in CONT; n=10 in 3-NOP

Table S9. Discriminant plasma metabolites identified by untargeted LC-ToF/MS in 14 weeks old calves, as affected by dietary supplementation of 3-NOP

Metabolite	VIP	Rt (min)	FC[1]	P-value	ID level[2]	Ion species	Exact mass	Formula	Δ m/z[3] (ppm)
Methionine	2.47	3.5	0.84	0.024	1	[M+H]+	150.0586	C5 H12 O2 N S	1.840
Leu/isol	6.96	3.6	0.93	0.082	1	[M+H]+	132.1020	C6 H14 O2 N	0.940
Tyrosine	2.31	3.6	0.87	0.020	1	[M+H]+	182.0812	C9 H12 O3 N	0.410
Phenylalanine	2.16	9.7	0.94	0.056	1	[M+H]+	166.0862	C9 H12 O2 N	-0.070
Tryptophane	3.14	10.3	0.89	0.151	2	[M+H]+	205.0969	C11 H13 O2 N2	-1.191
Glycocholic acid	6.34	13.3	0.49	0.076	2*	[M+H]+	466.3160	C26 H44 O6 N	-0.696
	1.22	13.3	0.36	0.082		[M+NH4]+	483.3430	C26 H47 O6 N2	0.365
	1.65	13.3	0.50	0.088		[M+Na]+	488.2979	C26 H43 O6 N Na	-0.838
	3.59	14.4	0.56	0.014		[M+H-3H20]	355.2626	C24 H35 O2	-1.624
	6.38	14.4	0.54	0.015		[M+H-2H2O]	373.2732	C24 H37 O3	-1.504
	2.45	14.4	0.54	0.015		[M-H2O+H]+	391.2836	C24 H39 O4	-1.549
	1.00	14.4	0.67	0.133		[M+NH4]	426.3209	C24 H44 O5 N	-1.290
Cholic acid	1.94	14.4	0.62	0.022	2*	[M+Na]	431.2764	C24 H40 O5 Na	-1.010
Lyso PC(16:1)	1.13	15.6	0.82	0.217	2	[M+H]+	494.3239	C24 H49 O7 N P	-0.436
Lyso PE(18:2)	1.76	15.9	0.76	0.052	2	[M+H]+	478.2925	C23 H45 O7 N P	-0.660

[1] Fold change ratio of the average vlue of treated to that of control.
[2] Identification level: 1 based on retention time, accurate mass and fragmentation pattern compared to analytical standard; 2 obtained by quering in-house database: based on retention time and accurate mass match, and for 2*also adducts pattern.
[3] Mass error, measured relatively to the theoretical exact mass.
