# Supplementary material for: Early life dietary intervention in dairy calves results in a long-term reduction in methane emissions
Source: Sci Rep. 2021 Feb 4;11:3003. doi: 10.1038/s41598-021-82084-9 (PMC7862406; doi:10.1038/s41598-021-82084-9)
Supplement: Supplementary file 2 — Supplementary Figures. [file 41598_2021_82084_MOESM2_ESM.pdf]

Supplementary Material - FIGURES

Early life intervention in dairy calves results in a long term reduction in methane emissions

S.J. Meale, M. Popova, C. Saro, C. Martin, A. Bernard, M. Lagree, D.R. Yáñez-Ruiz, H. Boudra, S. Duval, D.P. Morgavi

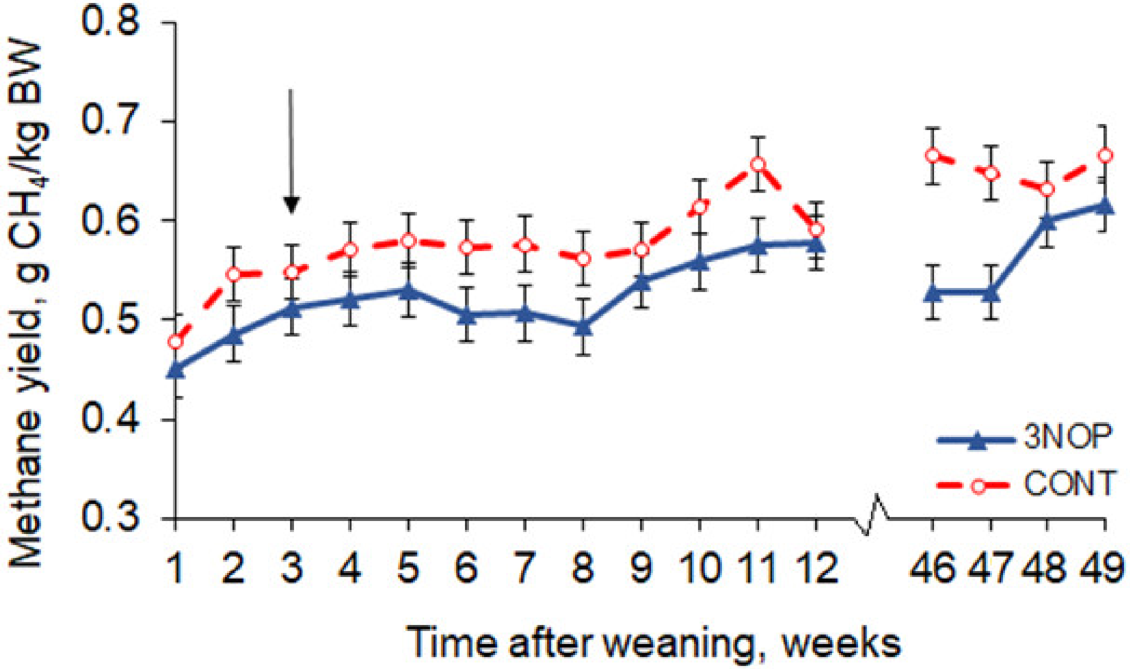

Fig. S1. Methane yield (g CH_4/kg BW) from calves fed CONT or 3-NOP diets in the post-weaning period. The arrow represents the end of the treatment period. Data represent means per week and SE bars (n=18). A tendency (p=0.08) for lower CH_4 yield in the post-weaning period was observed between CONT and 3-NOP calves.

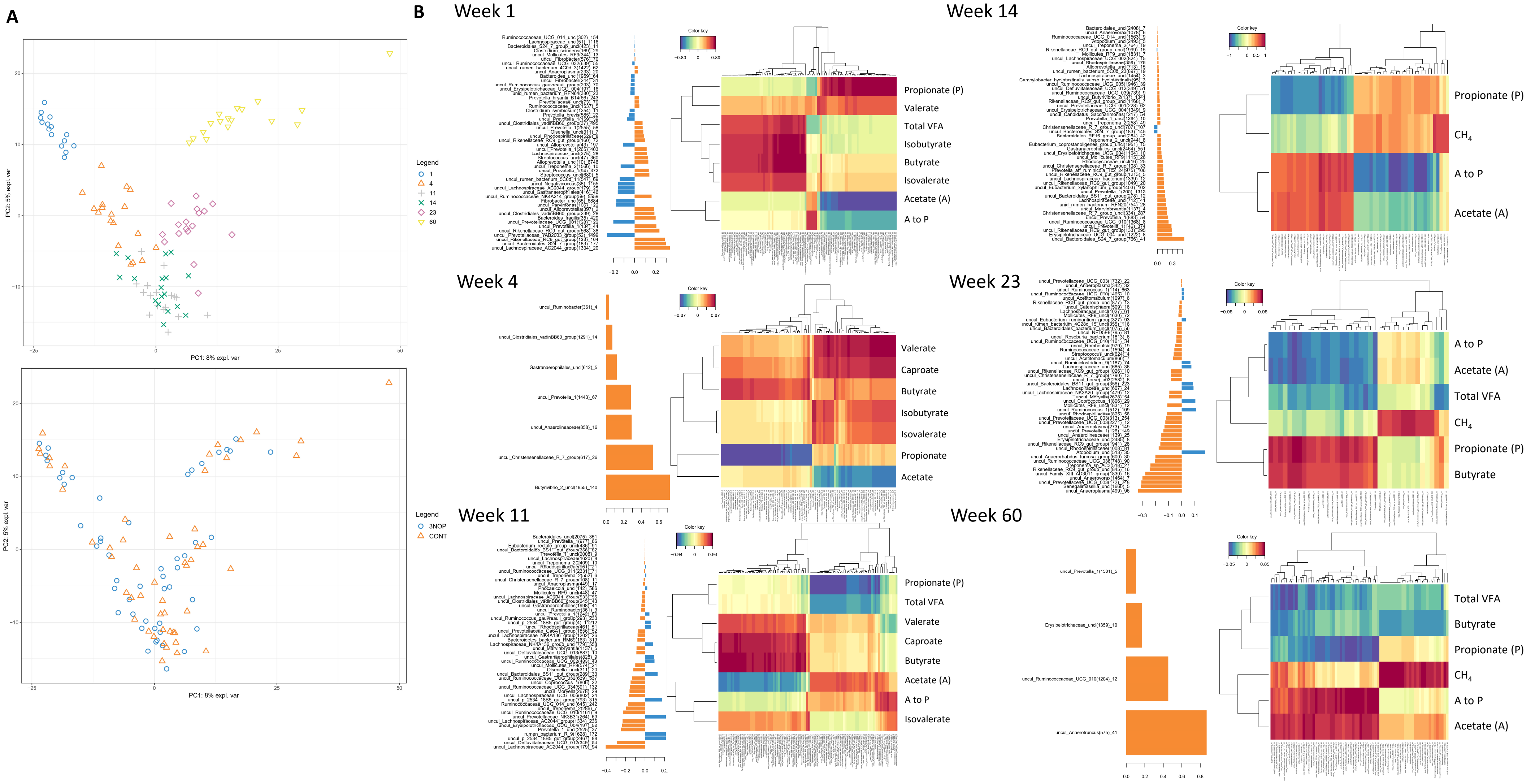

Fig. S2. Effect of 3-NOP supplementation on rumen Bacterial community in dairy calves up to 60 weeks of age. PCA plots of all weeks and treatments (A); discriminant OTUs explaining the separation in the first component loadings, CONT = orange and 3-NOP calves = blue (see Fig. 2 in the main text), and heatmap correlation (threshold value= 0.7) between discriminant OTUs and rumen fermentation parameters (B).

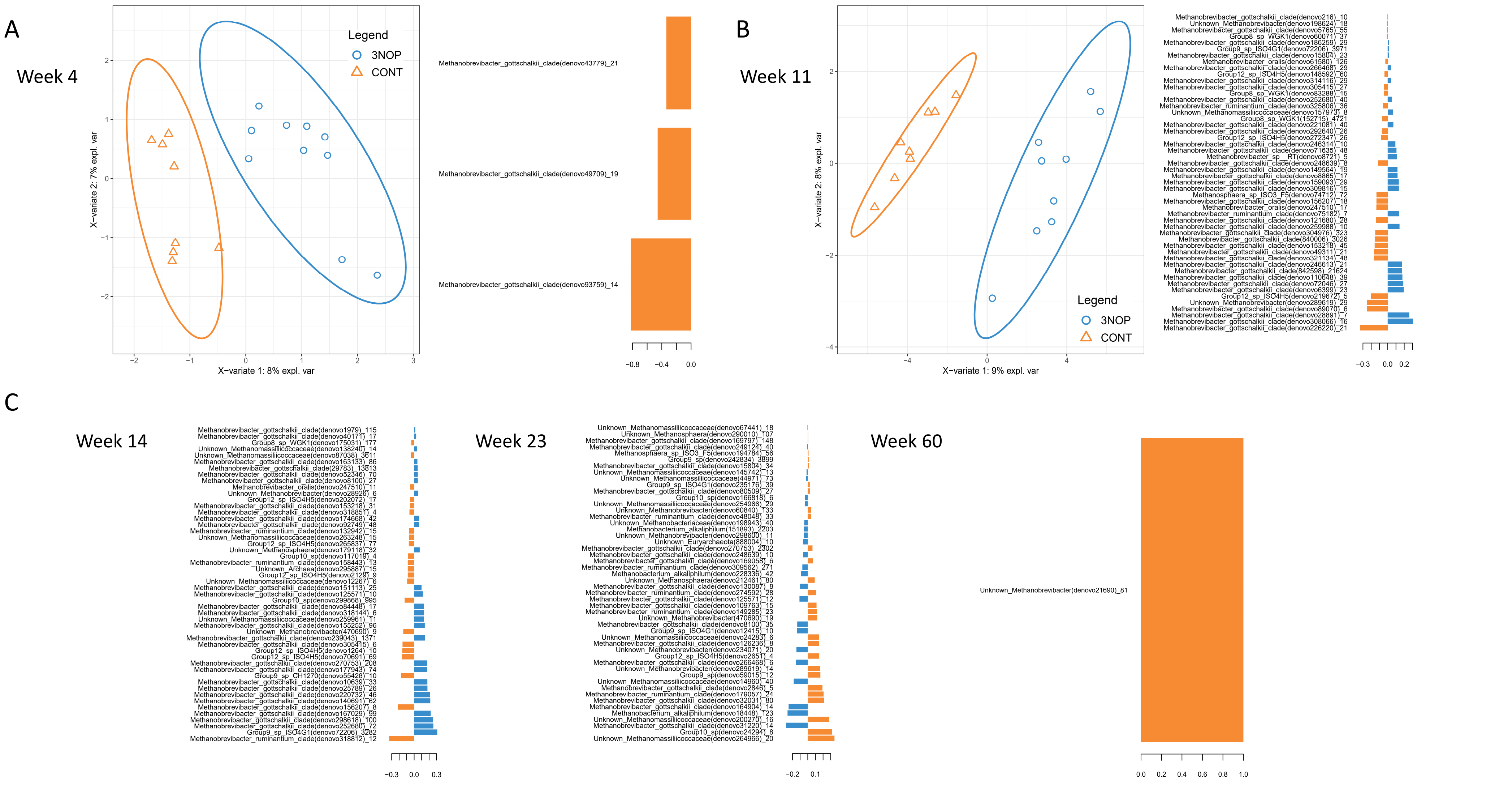

Fig. S3. Effect of 3-NOP supplementation on rumen Archaeal community in dairy calves. s-PLS-DA loading weights plots and loadings of discriminant OTUs in the first component of archaeal community structure in week 4 (A) and 11 (B); loadings of discriminant OTUs of archaeal community in calves aged 14, 23 and 60 weeks (s-PLS-DA plots showed in Fig. 3 B)(C).

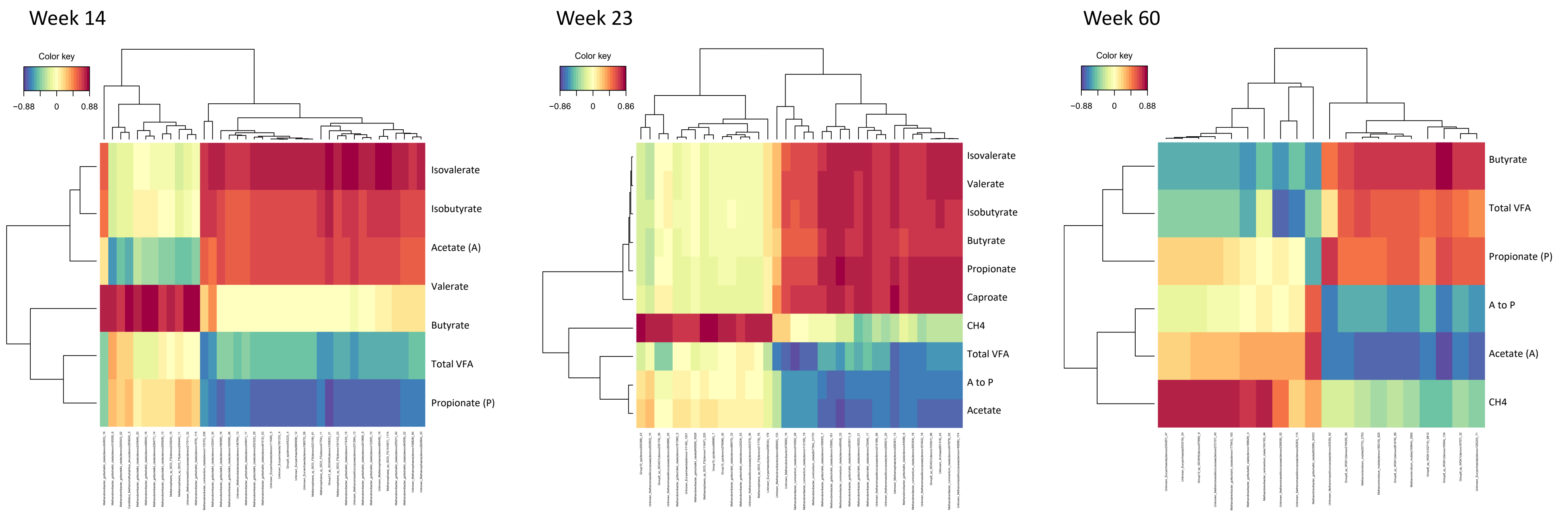

Fig. S4. Effect of 3-NOP supplementation on rumen Archaeal community in dairy calves. Heatmaps of discriminants OTUs correlated (threshold value= 0.7) to rumen fermentation parameters and methane emissions in calves aged 14, 23 and 60 weeks

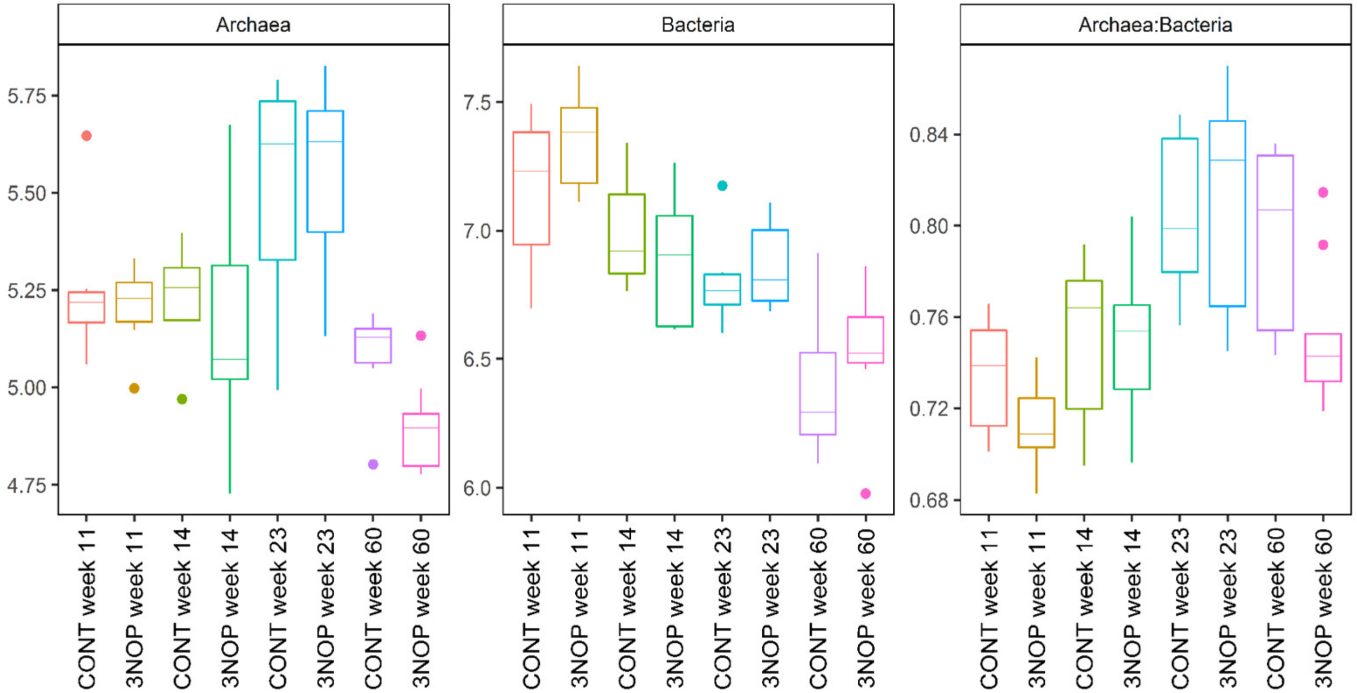

Fig. S5. Total Archaea and Bacteria, and Archaea to Bacteria ratio determined by qPCR (log_{10} copies/mL). No differences were observed between treatments within each week.

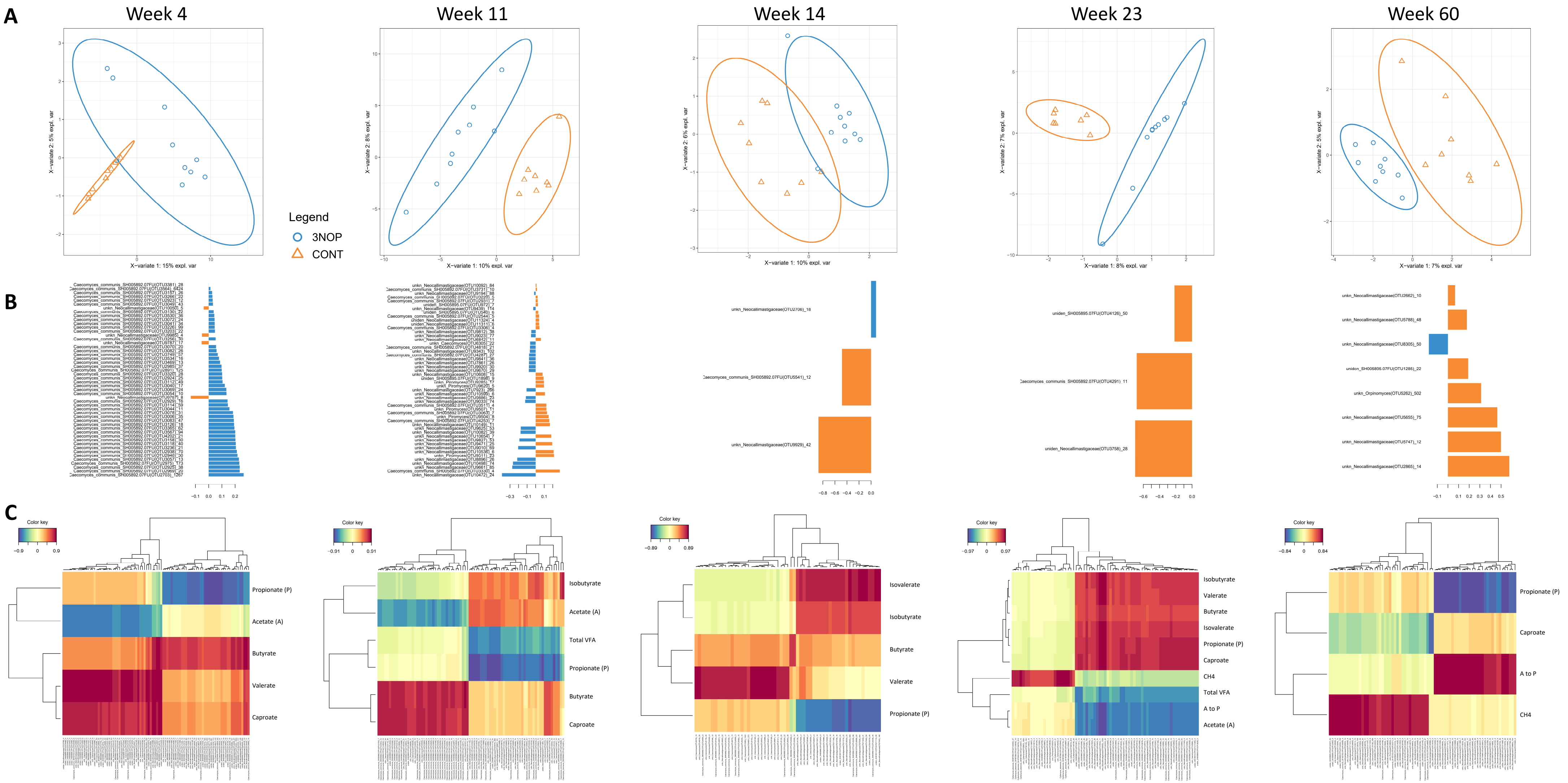

Fig. S6. Effect of 3-NOP supplementation on rumen anaerobic fungal community in dairy calves up to 60 weeks of age. s-PLS-DA loading weights plots of fungal community structure (A), loadings of discriminant OTUs (B) and heatmaps of discriminant OTUs versus rumen functional parameters (threshold value= 0.7) in weeks 4, 11, 14, 23 and 60 (C).

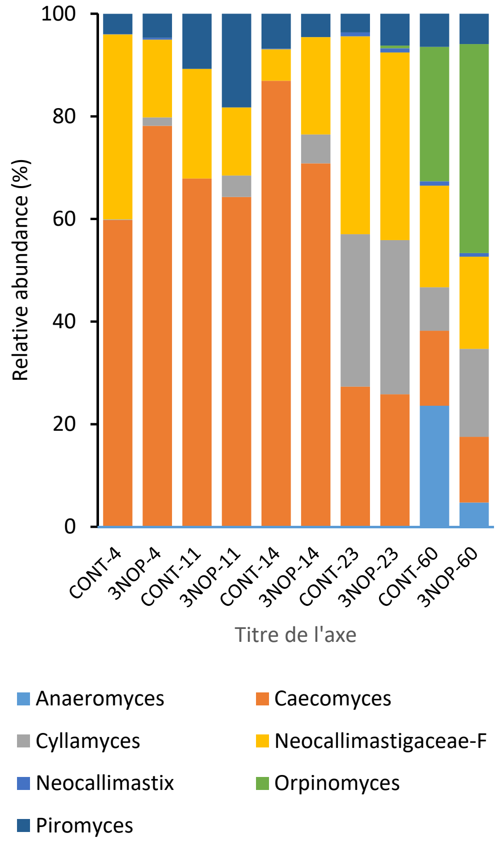

Fig. S7. Effect of 3-NOP supplementation on rumen anaerobic fungal genera in dairy calves. Control (CONT) and 3-NOP groups in weeks 4, 11, 14, 23 and 60.

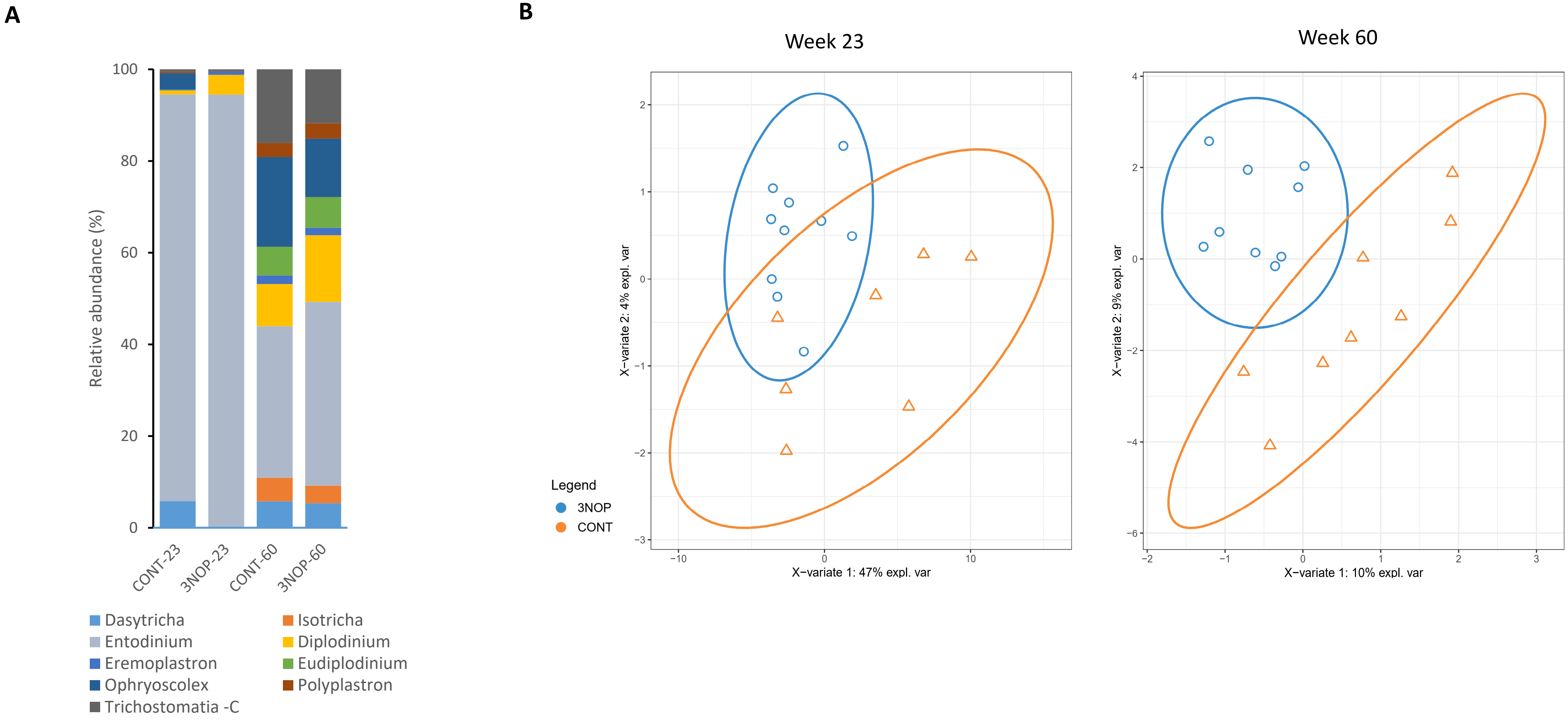

Fig. S8. Effect of 3-NOP supplementation on rumen protozoa in dairy calves at 23 and 60 weeks of age. Relative abundance of protozoa species (A). s-PLS-DA loading weights plots of protozoa community structure (B) showing no separation between groups.

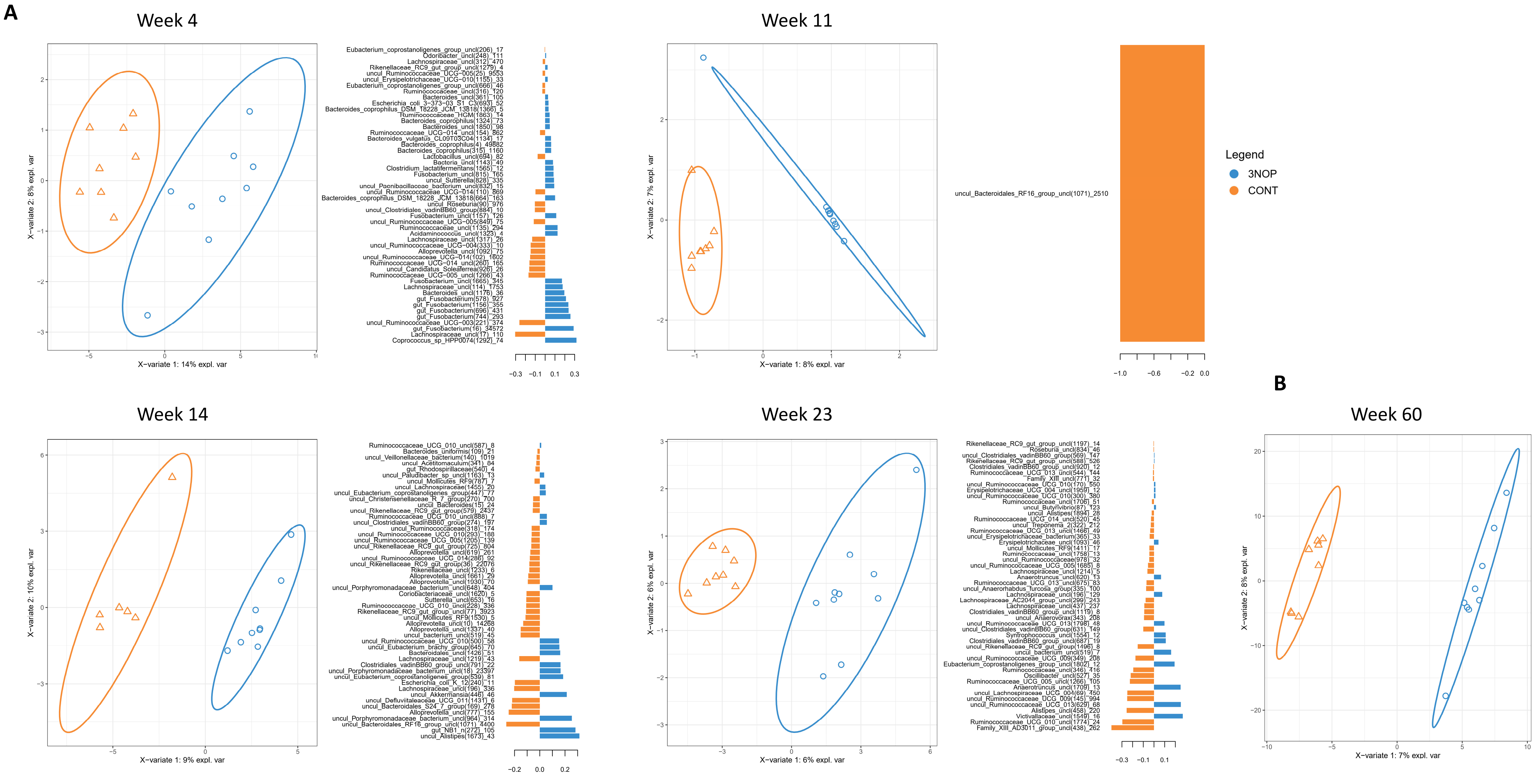

Fig. S9. Effect of 3NOP supplementation on faecal bacterial community in dairy calves up to 60 weeks of age. (A) s-PLS-DA loading weights plots of bacterial community structure and loadings of discriminant OTUs from week 4 to 23, (B) PLS-DA loading weights plot of week 60

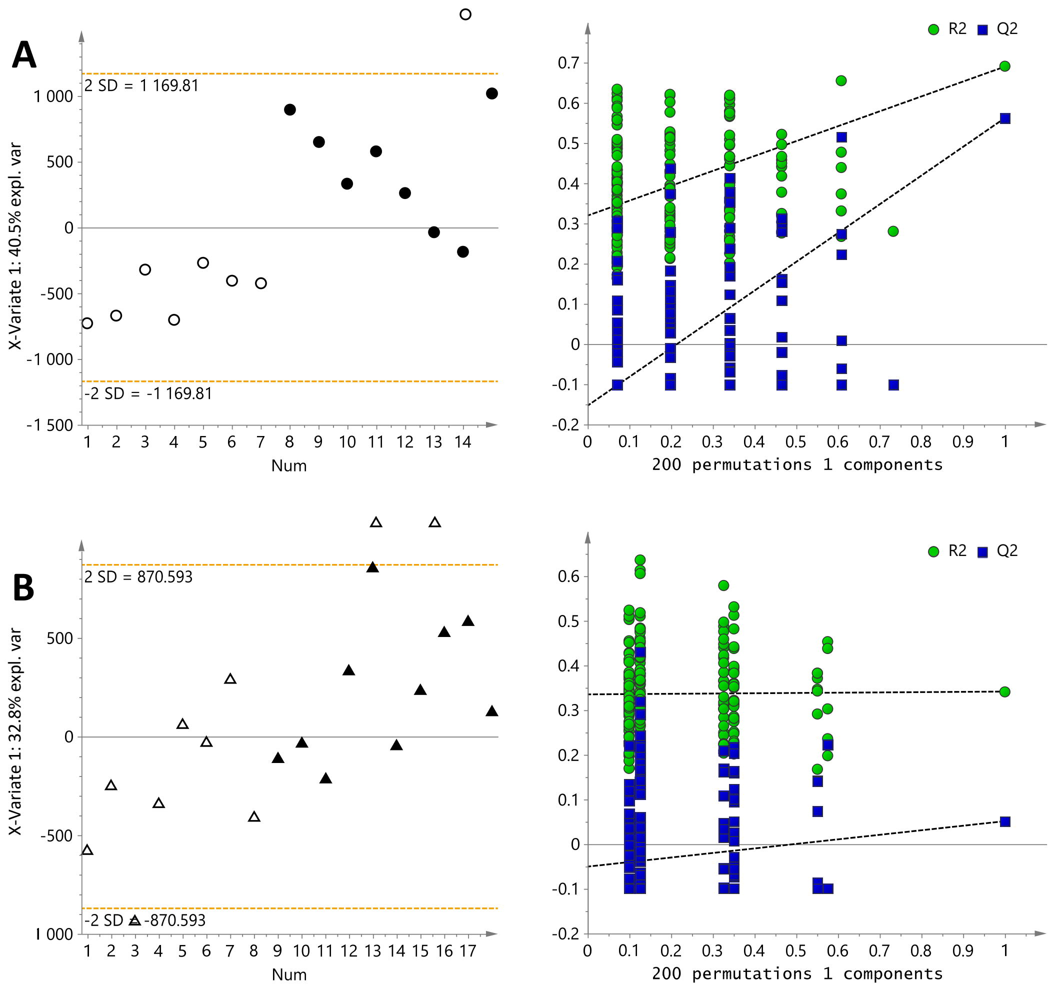

Figure S10. PLS-DA score plots with cross-validation of plasma metabolites from calves fed CONT or 3-NOP diets in week 14 (A) and in week 23 (B) following variable selection based on t-test P<0.3. PLS-DA models had only one component indicated on the y axis whereas calves are distributed on the x axis. The right panel shows permutation tests for week 14: R^2=0.69; Q^2=0.56 (upper side) and for week 23: R^2=0.34; Q^2=0.05 (lower side). Results of R^2 (goodness of fit) and Q^2 (goodness of prediction) of permuted models must be lower than the original values which validates the model at week 14 but not that at week 23.

Data were grouped according to CON (white) and 3NOP calves (dark). One control calf could not be sampled (CON= 7). In week 14, two calves in the 3NOP group were considered outliers and not included in the analysis.

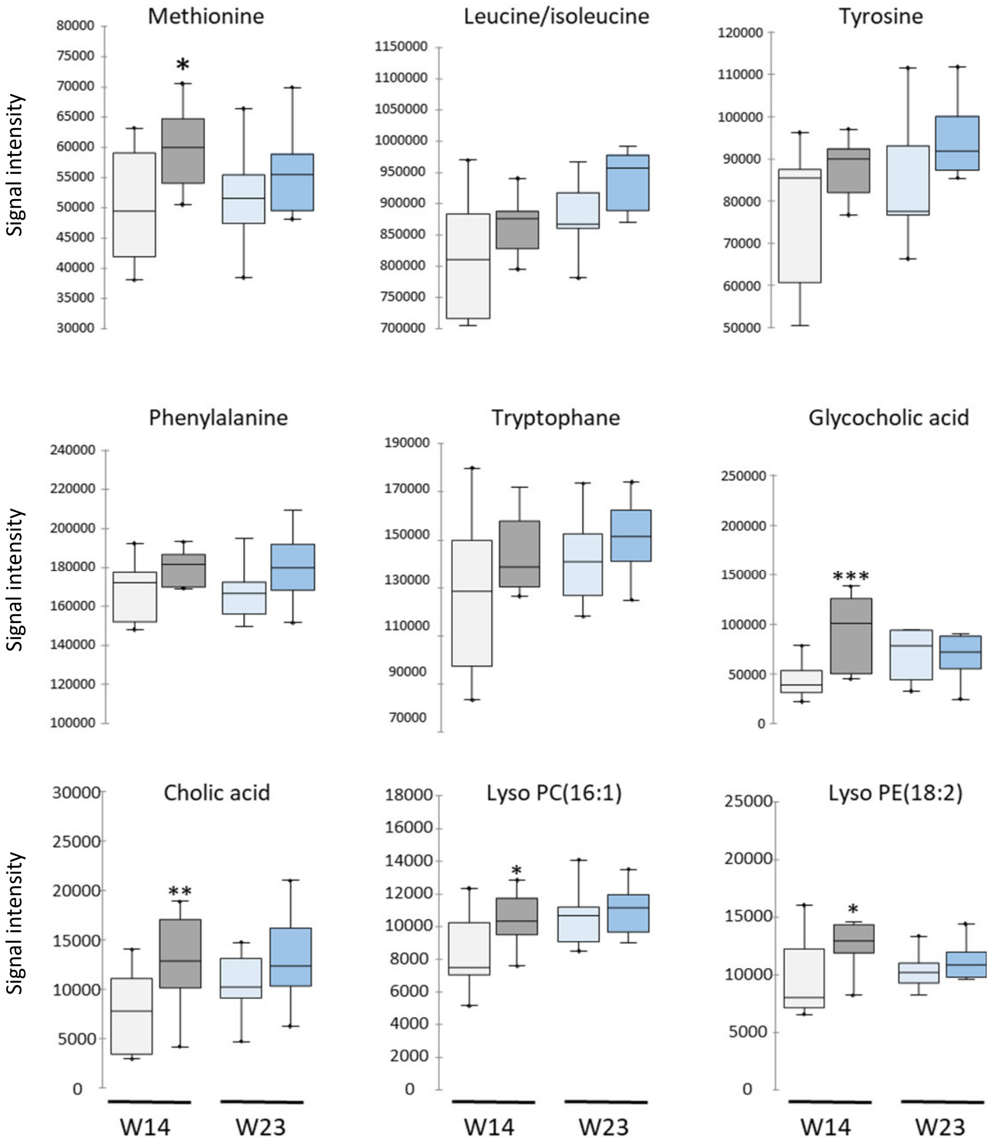

Figure S11. Plasma concentration of nine metabolites from calves fed CONT or 3NOP diets in the post-weaning period at week 14 and 23. At week 14 these metabolites were discriminant in the PLS-DA analysis. * $P < 0.1$; ** $P < 0.05$; *** $P < 0.01$ ANOVA test: CONT (light colours) compared to 3-NOP (dark colours) within week.
